# ﻿Intraspecific divergence of diploid grass *Aegilopscomosa* is associated with structural chromosome changes

**DOI:** 10.3897/CompCytogen.17.101008

**Published:** 2023-04-12

**Authors:** Ekaterina D. Badaeva, Violetta V. Kotseruba, Andnrey V. Fisenko, Nadezhda N. Chikida, Maria Kh. Belousova, Peter M. Zhurbenko, Sergei A. Surzhikov, Alexandra Yu. Dragovich

**Affiliations:** 1 N.I.Vavilov Institute of General Genetics, Russian Academy of Sciences, Gubkina str. 3, GSP-1, Moscow 119991, Russia Engelhardt Institute of Molecular Biology, Russian Academy of Sciences Moscow Russia; 2 Engelhardt Institute of Molecular Biology, Russian Academy of Sciences, Vavilova str. 32, GSP-1, Moscow 119334, Russia N.I.Vavilov Institute of General Genetics, Russian Academy of Sciences Moscow Russia; 3 Komarov Botanical Institute, Russian Academy of Sciences, Prof. Popova str. 2, Saint Petersburg 197376, Russia Komarov Botanical Institute, Russian Academy of Sciences Saint Petersburg Russia; 4 N.I. Vavilov Institute of Plant Genetic Resources (VIR), Ministry of Science and Higher Education, Bolshaya Morskaya str. 42-44, Saint Petersburg 190000, Russia N.I. Vavilov Institute of Plant Genetic Resources (VIR), Ministry of Science and Higher Education Saint Petersburg Russia

**Keywords:** *Aegilopscomosa*, *Ae.c.comosa*, *Ae.c.heldreichii*, electrophoresis, Fluorescence in situ hybridization (FISH), intraspecific diversity, karyotype, repetitive DNA probes, seed storage proteins (gliadins)

## Abstract

*Aegilopscomosa* Smith in Sibthorp et Smith, 1806 is diploid grass with MM genome constitution occurring mainly in Greece. Two morphologically distinct subspecies – *Ae.c.comosa* Chennaveeraiah, 1960 and *Ae.c.heldreichii* (Holzmann ex Boissier) Eig, 1929 are discriminated within *Ae.comosa*, however, genetic and karyotypic bases of their divergence are not fully understood. We used Fluorescence in situ hybridization (FISH) with repetitive DNA probes and electrophoretic analysis of gliadins to characterize the genome and karyotype of *Ae.comosa* to assess the level of their genetic diversity and uncover mechanisms leading to radiation of subspecies. We show that two subspecies differ in size and morphology of chromosomes 3M and 6M, which can be due to reciprocal translocation. Subspecies also differ in the amount and distribution of microsatellite and satellite DNA sequences, the number and position of minor NORs, especially on 3M and 6M, and gliadin spectra mainly in the a-zone. Frequent occurrence of hybrids can be caused by open pollination, which, along with genetic heterogeneity of accessions and, probably, the lack of geographic or genetic barrier between the subspecies, may contribute to extremely broad intraspecific variation of GAA_n_ and gliadin patterns in *Ae.comosa*, which are usually not observed in endemic plant species.

## ﻿Introduction

*Aegilopscomosa* Smith ex Sibthorp et Smith, 1806 is annual diploid grass (2n=2x=14) with the MM genome constitution, which grows mainly in coastal and inland Greece, rarely – in coastal regions of Albania and Former Yugoslavia (Zhukovsky 1928; Eig 1929; Van Slageren 1994; Kilian et al. 2011). Several scattered populations have been found in Turkey (Zhukovsky 1928; Van Slageren 1994). Recently *Ae.comosa* was discovered also in Cyprus and Bulgaria (Van Slageren 1994).

Two morphologically distinct forms are discriminated within *Ae.comosa*; usually they are treated as subspecies of *Ae.comosa*: subsp. comosa Chennaveeraiah, 1960, thereafter *comosa*, and subsp. heldreichii (Holzmann et Boissier) Eig, 1929 thereafter *heldreichii* (Eig 1929; Hammer 1980; Kilian et al. 2011). Some taxonomists however recognize them as two distinct species: *Ae.comosa* and *Ae.heldreichii* (Boissier) Holzmann 1884 (Zhukovsky 1928; Chennaveeraiah 1960; Boguslavsky and Golik 2003), or as varieties of *Ae.comosa* [var. comosa Boissier, 1884 and var. subventricosa Jaubert et Spach ex Bornmüller, 1898 (Van Slageren 1994)]. Subspecies of *Ae.comosa* grow together, often in a mix with *Ae.caudata* Linneaus, 1753 on roadsides, grasslands and hillsides, sometimes in cultivated fields (Zhukovsky 1928). Except for Greece, *Ae.comosa* is uncommon to rare throughout its range.

Based on morphological similarity of *Ae.comosa* (both *comosa* and *heldreichii*) with *Ae.uniaristata* Visiani, 1852, P. Zhukovsky (1928) placed them into a common section Comopyrum Zhukovsky, 1928. These species however are genetically distinct and carry different types of nuclear and cytoplasmic genomes – M and N, respectively (Kimber et al. 1983; Kimber and Tsunewaki 1988). Radiation of *Ae.comosa* and *Ae.uniaristata* was accompanied by different structural chromosomal rearrangements (Molnár et al. 2016; Li et al. 2021; Said et al. 2021), which led to significant karyotype divergence of these species (Chennaveeraiah 1960; Teoh and Hutchinson 1983; Badaeva et al. 1996a, 2011; Friebe et al. 1996; Iqbal et al. 2000; Song et al. 2020; Li et al. 2021). Subspecies comosa and *heldreichii* are characterized by similar karyotype structures (Chennaveeraiah 1960), however meiotic analysis of their F_1_ hybrid showed that they differ by one reciprocal translocation (Kihara 1940).

C-banding proved to be effective tool in phylogenetic analyses of the Triticeae. This method was employed to characterize karyotypes of all diploid and polyploid *Aegilops* Linneaus, 1753 species including *Ae.comosa* (Teoh and Hutchinson 1983; Friebe et al. 1992, 1993; Friebe et al. 1996; Badaeva et al. 1998, 2002, 2004), and differences between subspecies comosa and *heldreichii* in the amount and distribution of C-bands have been reported (Teoh et al. 1983; Friebe et al. 1996). On the contrary, other researchers failed to discriminate *comosa* from *heldreichii* using FISH with GAA_n_ microsatellite probe, which produces C-banding-like pattern (Song et al. 2020).

In earlier classifications C-banded *Ae.comosa* chromosomes were arranged in a decreasing length and designated with capital letters A – G (Teoh et al. 1983; Georgiou et al. 1992). First genetic nomenclature of the M-genome chromosomes was developed by B. Friebe et al. (1996) based on similarities of their C-banding patterns with homoeologous chromosomes of other *Aegilops* species. This system was later proved by chromosome sorting and single-gene FISH (Molnár et al. 2016; Said et al. 2021). Some controversy remained in classifying chromosomes 2M and 5M, which are indistinguishable by flow sorting due to similar morphology and same DNA content. Even more discrepancies in chromosome designations exist with the nomenclature developed by C. Liu et al. (2019) on the basis of analysis of addition and substitution wheat-*Ae.comosa* lines using FISH and PLUG markers. Thus, chromosome classification of this species still needs verification.

*Aegilopscomosa* plays an important role in the evolution of polyploid *Aegilops*. Based on “analyzer” method H. Kihara (Kihara 1947, 1954) hypothesized that it gave rise to five tetraploid *Aegilops* species (bold **M** indicates genome modification): *Ae.crassa* Boissier, 1846 (DD**MM**), *Ae.columnaris* Zhukovsky, 1928 (UU**MM**), *Ae.neglecta* Requien ex Bertoloni, 1834 (UU**MM**), *Ae.biuncialis* Visiani, 1842 (UU**MM**), and *Ae.geniculata* Roth, 1787 (UU**MM**). Recent studies, however, did not confirm the presence of the M-genome in *Ae.crassa*, *Ae.columnaris* and *Ae.neglecta* (Resta et al. 1996; Badaeva et al. 2004; Edet et al. 2018; Abdolmalaki et al. 2019), but it was proved for *Ae.biuncialis* and *Ae.geniculata* (Kihara 1954; Kimber et al. 1988; Resta et al. 1996; Tsunewaki 1996; Friebe et al. 1999; Badaeva et al. 2004; Molnár and Molnár-Láng 2010; Molnár et al. 2011; Abdolmalaki et al. 2019; Said et al. 2022). All these papers reported significant genome modifications in karyotypes of these two tetraploid species, which seemed to proceed differently in *Ae.biuncialis* and *Ae.geniculata* (Badaeva et al. 2004; Said et al. 2022). *Aegilopscomosa* as well as its tetraploid derivatives exhibited an extremely broad intraspecific variation of C-banding and/ or GAA_n_ labeling patterns (Teoh et al. 1983; Georgiou et al. 1992; Friebe et al. 1996; Badaeva et al. 2004; Song et al. 2020), which may impede delimitation of taxa boundaries and tracking evolutionary changes in karyotype of polyploids using these markers.

Among a broad range of botanical, cytogenetic, biochemical and molecular markers employed for evaluating intraspecific and interspecific diversity of wild and cultivated plant species, seed storage proteins (gliadins) appear to be relatively cheap, but informative markers for polymorphism analysis. Gliadins (Gli) belong to protein fraction prolamines, which is characterized by high glutamine and proline amino acid content and by specific molecular structure (size, domen composition, biochemical properties) (Shewry and Halford 2002). Electrophoretic spectra of gliadins allow discrimination, with high effectiveness, of lines, cultivars and varieties of tetraploid and hexaploid wheat; gliadin profiles are used to assess samples’ heterogeneity and to evaluate phylogenetic relationships between species and accessions (Metakovsky et al. 2018). So far, polymorphism analyses of *Aegilops* based on gliadin loci were mainly focused on *Ae.tauschii* Cosson, 1850, the D-genome donor of common wheat (Yan et al. 2003; Dudnikov 2018; Badaeva et al. 2019a), and only few publications dealt with other *Aegilops* species (Cole et al. 1981; Medouri et al. 2015; Garg et al. 2016). On the other hand, owing to similar structure of gliadin loci in wheat and *Aegilops* (Dong et al. 2016; Huo et al. 2018) and extremely high polymorphism, gliadins can serve as supplementary markers for the assessing genetic variability of *Ae.comosa* and clarifying phylogenetic relationships between the subspecies.

*Aegilopscomosa* possesses a number of agronomically valuable traits such as pest and disease resistance (Riley et al. 1968; Gill et al. 1985; Boguslavsky and Golik 2003) and salt tolerance (Xu et al. 1996), which can potentially be used for wheat improvement. In late 60^th^ of XX R. Riley, V. Chapman, and R.O.Y.Johnson introduced resistance to yellow rust from *Ae.comosa* into wheat cultivar “Compare” by genetically induced homoeologous recombination (Riley et al. 1968). Several wheat-*Ae.comosa* amphiploid and introgression lines have been developed in China and UK (Weng 1995; Liu et al. 2019; Zuo et al. 2020); some of these lines showed good resistance to yellow rust and powdery mildew (Liu et al. 2019). However, modifications of the M-genome chromosomes over the course of evolution, in particular, species-specific translocations, prevent the direct utilization of *Ae.comosa* gene pool in wheat breeding (Nasuda et al. 1998). Manipulations with genetic material of *Ae.comosa* require a deeper understanding of the genome of this species, its chromosomal structure and the range of polymorphism.

The aim of our study was a comparative analysis of Ae.comosasubsp.comosa and subsp. heldreichii on a broad sample of accessions of different geographical origins using FISH with fifteen DNA probes and electrophoretic analysis of seed storage proteins (gliadins) in order to characterize polymorphism and reveal mechanisms leading to divergence of subspecies.

## ﻿Material and methods

### ﻿Material

**Figure 1.**
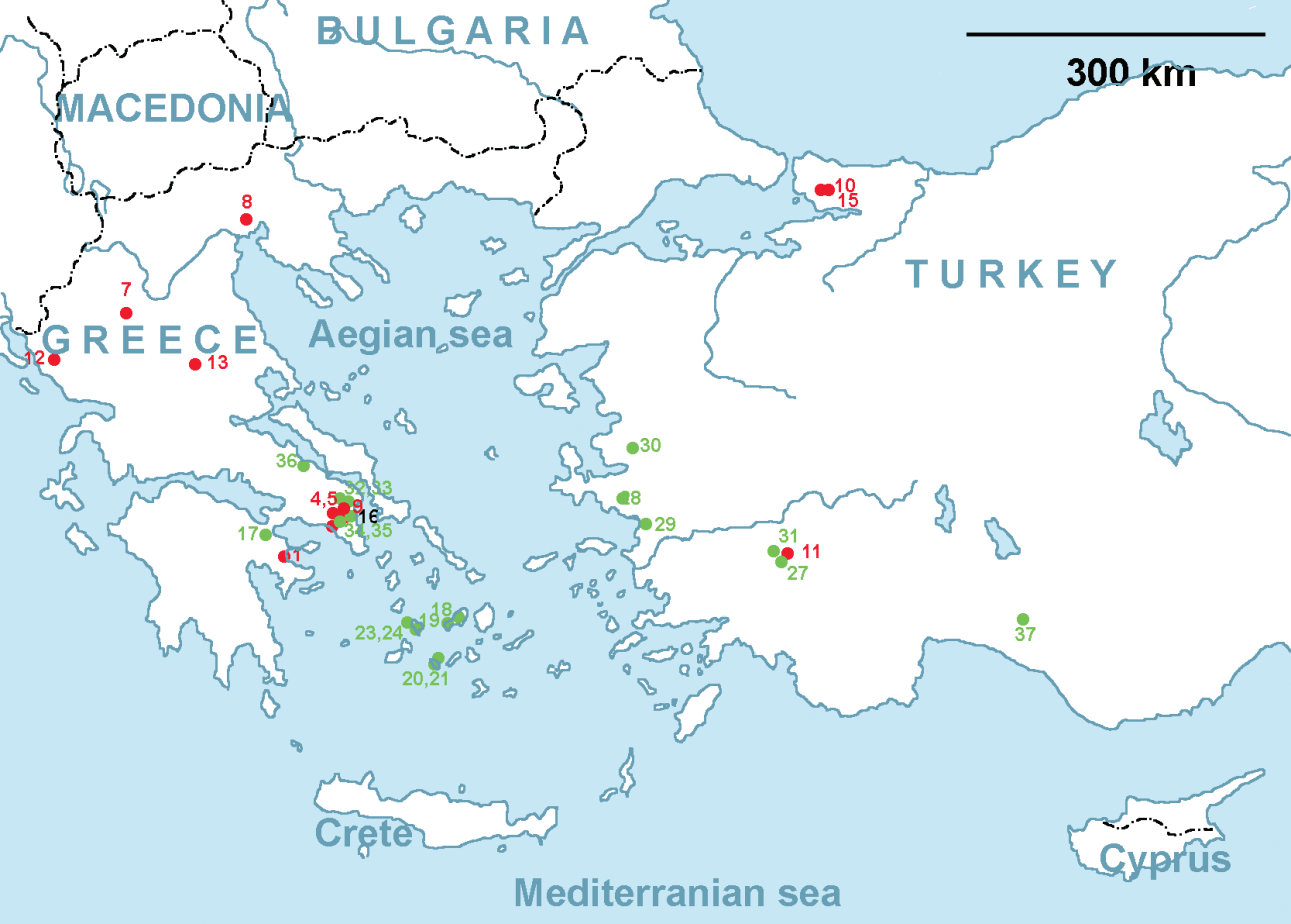
Geographical location of Ae.comosasubsp.comosa (green dots) and Ae.comosasubsp.heldreichii (red dots) accessions with known collection sites. The green ( subsp. comosa) and red (subsp. heldreichii) numerals specify the accession numbers according to Suppl. material 9.

**Figure 2.**
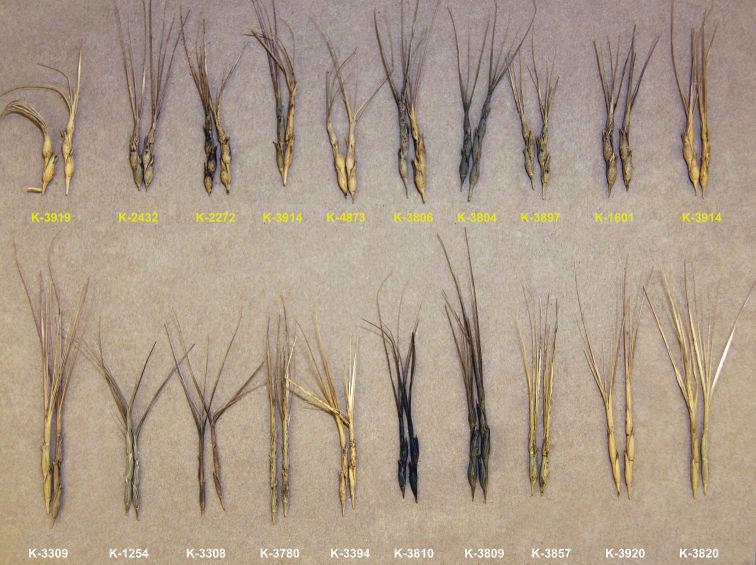
Comparison of spike morphology of *Ae.comosaheldreichii* (top raw) and *comosa* (bottom raw).

Thirty-six accessions of *Ae.comosa* including 20 accessions of *comosa* and 16 accessions of *heldreichii* collected from different regions of Greece and Turkey (Fig. 1) and maintained in genetic collections of N. I. Vavilov All-Russian Institute of Plant Genetic Resources (VIR), St.-Petersburg, Russia, and Leibniz Institute of Plant Genetics and Crop Plant Research (IPK), Gatersleben, Germany were used in our study (Suppl. material 9). Accessions of *comosa* and *heldreichii* show clear differences in spike morphology (Fig. 2).

Thus, spikes of subsp. comosa plants are slender, narrowly cylindrical, tapering toward apex, with 3–4 fertile and 0–2 rudimentary spikelets. Glumes of lateral spikelets have one tooth and one short awn, the apical spikelet has three well-developed awns, the central one of 4–11 cm long and lateral – 2.5–3.5 cm long. Spikes of subsp. heldreichii plants are shorter and stouter, not or hardly tapering toward the apex, with one rudimentary and 1–3 fertile spikelets. Lateral spikelets are urceolate, the apical one is obconical. Glumes are ovate, the lateral glumes with broadly triangular tooth on abaxial site and short awn on adaxial side. Apex of apical glume extends into three 3–3.5 cm-long cetulose awns. Lateral awns are shorter and more slender, often reduced to teeth or even absent (Van Slageren 1994). Accessions of both subspecies significantly vary in spike length and color (Fig. 2), and accession K-3809 (subsp. comosa) is characterized by longest spike with black color.

### ﻿DNA probes

Fifteen oligo-probes were used in FISH analysis. Microsatellite probes were labeled with either 6-FAM (GTT_10_, GAA_10_) or Cy3/TAMRA (GAA_10_, ACT_10_, AC_20_) from the 5’-end. Oligo-18S was designed based on conservative region of the 18S rRNA gene. Melting temperature and potential secondary structures were calculated using OligoCalc (Kibbe 2007). Names and nucleotide sequences of other probes are listed in Table 1. The probes GTT_10_, oligo-42, oligo-44, oligo-45, oligo-18SrDNA (o-18S), oligo-pSc119.2 were synthesized in Evrogen (Moscow, Russia); GAA_10_-FAM, pTa-71-2 (pTa71), oligo-pTa-794 (5SrDNA), oligo-k566 were synthesized in Syntol (Moscow, Russia); ACT_10_, GAA_10_-Cy3, oligo-pAs1-1 (pAs1), oligo-pTa-713, oligo-pTa-535 were synthesized in the Laboratory of Biological Microchips at the Engelhardt Institute of Molecular Biology, Moscow, Russia.

**Table 1.** Oligo-probes used in FISH analysis.

Probe name	Sequence	Amount of probe (ng/ slide)	Reference
Oligo-pTa-71	FAM/5’- GGG CAA AAC CAC GTA CGT GGC ACA CGC CGC CTA-3’	21.1	Tang et al. 2014
Oligo-18S	FAM/5’- CTC GGA TAA CCG TAG TAA TTC TAG AGC TAA TAC GTG CAA CAA ACC CCG-3’	40.5	Current paper
Oligo-5S rDNA	Cy3/5’-TCA GAA CTC CGA AGT TAA GCG TGC TTG GGC GAG AGT AGT AC-3’	27.1	Yu et al. 2019
Oligo-GAA_n_	TAMRA (or FAM)/5’-GAA GAA GAA GAA GAA GAA GAA GAA GAA GAA-3’	21,4	Cuadrado et al. 2008a
Oligo-GTT_n_	FAM/5’-GTT GTT GTT GTT GTT GTT GTT GTT GTT GTT-3’	19.5	Cuadrado et al. 2008a
Oligo-ACT_n_	Cy3/5’-ACT ACT ACT ACT ACT ACT ACT ACT ACT ACT-3’	20.1	Cuadrado et al. 2008a
Oligo-AC	TAMRA/5’-AC AC AC AC AC AC AC AC AC AC AC AC AC AC AC AC AC AC AC AC AC AC AC AC-3’	18.4	Cuadrado et al. 2008a
Oligo-pSc119.2	FAM/5’- CCG TTT TGT GGA CTA TTA CTC ACC GCT TTG GGG TCC CAT AGC TAT -3’	28.3	Tang et al. 2014
Oligo-pAs1	Cy3/5’-CCT TTC TGA CTT CAT TTG TTA TTT TTC ATG CAT TTA CTA ATT ATT TTG AGC TAT AAG AC-3’	36.7	Tang et al. 2014
Oligo-pTa-713	Cy3/5’- GTC GCG GTA GCG ACG ACG GAC GCC GAG ACG AGC ACG TGA CAC CAT TCC CAC CCT GTC TA-3’	37.9	Tang et al. 2016
Oligo-pTa-535	Cy3/5’- AAA AAC TTG ACG CAC GTC ACG TAC AAA TTG GAC AAA CTC TTT CGG AGT ATC AGG GTT TC-3’	37.4	Tang et al. 2014
Oligo-k566	FAM/5’- ATC CTA CCG AGT GGA GAG CGA CCC TCC CAC TCG GGG GCT TAG CTG CAG TCC AGT ACT CG-3’	37.1	Tang et al. 2016
Oligo-45	TAMRA/5’-CGG CCG CTC CGC GCG TCG CCA TCG GTT GGT CAC CTC ATC ACC ACT-3’	28.2	Tang et al. 2018a
Oligo-42	FAM/5’-CTC GCT CGC CCA GCT GCT GCT ACT CCG GCT CTC GCT CGA TCG-3’	26.1	Tang et al. 2018a
Oligo-44	TAMRA/5’-TAG CTC TAC AAG CTA GTT CAA ATA ATT TTA CAC TAG AGT TGA AC-3’	27.88	Tang et al. 2018a

### FISH analysis

The seeds are germinated on moist filter paper in Petri dishes at 24 °C. The seedlings with ~0.5 cm roots are transferred into 1.25 mM solution of hydroxyurea for 18 h, washed thoroughly with distilled water and grown in Petri dishes with distilled water for 5 h, as described in (Badaeva et al. 2017). The roots are cut and pretreated in ice water for 24 h and then fixed in the solution of ethanol : glacial acetic acid (3 : 1). Fixed roots are kept in fixative solution at -20 °C until use.

Metaphase cells are prepared by squashing, coverslips are removed after freezing in liquid nitrogen, and slides are kept in 96% ethanol at -20 °C. Fluorescence in situ hybridization is carried out according to previously published protocol (Badaeva et al. 2017). The slides are examined on a Zeiss Imager D1 epifluorescent microscope. Metaphase plates are captured with a 100^x^ objective using black and white digital camera Axiocam HRm using a software AxioVision, release 4.8. The images are processed using Adobe Photoshop, version 7.0.

### ﻿Electrophoretic analysis of seed storage proteins (gliadins)

Electrophoresis (EP) in polyacrylamide gel (PAAG) according to the previously published protocol (Metakovsky and Novoselskaya 1991) was employed to obtain gliadin spectra of 26 accessions of *Ae.comosa*. Since no information on the composition and inheritance of the blocks of gliadin components in this species was available from literature, electrophoretic spectra of all accessions must be compared with an etalon sample with the known genetic control of components. In wheat and related species the gliadin spectrum of bread wheat cultivar Bezostaya-1 serves as an etalon (Novoselskaya-Dragovich et al. 2018).

The gliadin spectra of the Triticeae are traditionally divided into four zones, α, β, γ and ω-zones, depending on electrophoretic mobility of individual polypeptides (Woychik et al. 1961). Peptides from the ω-zone are coded by genes located on group 1 chromosomes, while those from the α-zone – by genes of group 6 chromosomes. Components from β and γ zones are controlled by chromosomes of both genetic groups (Dong et al. 2016; Huo et al. 2018). Based on this information we presume that electrophoretic components from the α-zone of the spectra of *Ae.comosa* accessions are encoded by 6M, whereas from the ω-zone, by 1M chromosome.

## ﻿Results

### ﻿Intraspecific diversity of *Ae.comosa* in karyotype structure

**Figure 3.**
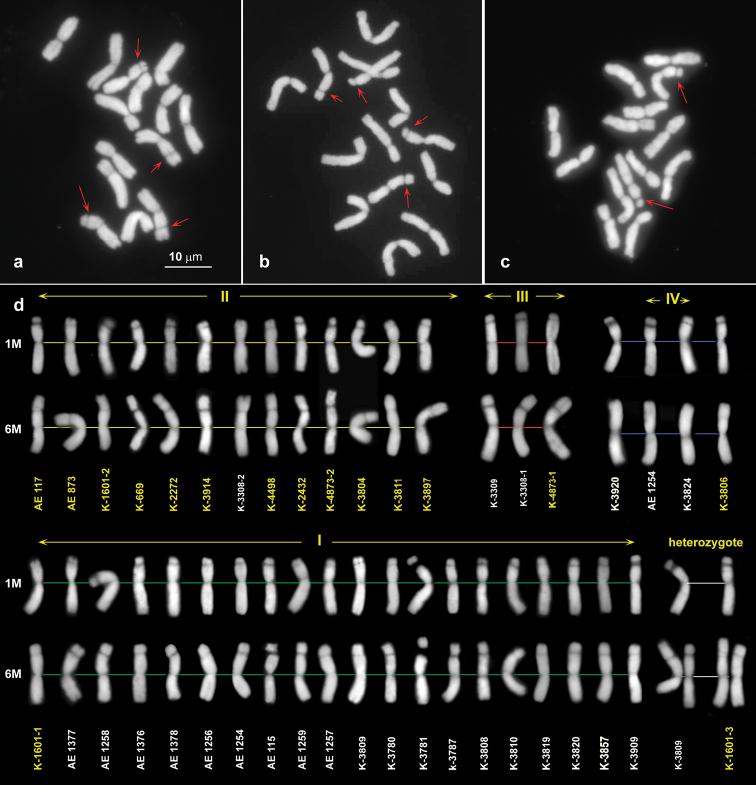
Metaphase cells of Ae.comosasubsp.heldreichii, AE 783 (**a**) and subsp. comosa, AE 1258 (**b**) and AE 1254 (**c**). Satellite chromosomes are shown with red arrows. (**d**) structural diversity of the SAT chromosomes in *Ae.comosa*: accessions of *comosa* are designated with white numbers, *heldreichii* – with yellow numbers. Scale bar: 10 µm.

Two subspecies (*comosa* and *heldreichii*) of *Ae.comosa* have similar karyotype structures, which include metacentric, submetacentric and subacrocentric chromosomes (Fig. 3 a–c). Despite overall karyotypic similarity, we observe variation in the number and morphology of satellite (SAT) chromosomes (Fig. 3d). Most accessions have two pairs of satellite chromosomes differing in morphology (Fig. 3a, b), but few genotypes carry only one SAT pair (Fig. 3c).

The satellite on one pair is always small, and this chromosome is classified as 1M. The satellite on the second pair – 6M, is much larger and appears on physically longer arm. Comparison of the SAT chromosomes allows to divide *Ae.comosa* accessions into four groups (Fig. 3d). Group I is characterized by long submetacentric chromosome 1M with a small SAT attached to the short arm. The SAT on 6M is very large and occupies nearly half of the arm length. Most accessions from this group belong to subsp. heldreichii, and this combination of satellite chromosomes is designated “*heldreichii*-like”. Group II differs from Group I in shorter satellite length (approximately 1/3 to 1/4 of the arm) on the chromosome 6M. Accessions from this group belong predominantly to subsp. comosa and we designate this combination of satellite chromosomes as “*comosa*-like”. Groups III and IV include representatives of both subspecies. Group III shows altered morphology of both SAT chromosome pairs, whereas Group IV contains just one pair of SAT chromosomes – 1M (K-3308, K-3309, K-4873; Fig. 3d). Group IV includes one of the three analyzed genotypes of AE 1254, one of the two genotypes of each K-3824, K-3920 (all *comosa*), and all K-3806 genotypes (*heldreichii*). All these genotypes are characterized by heteromorphic pair of 6M chromosomes: one homolog carries large, while the second – much smaller satellite on the long arms (Fig. 3, “heterozygote”), this group presumably represents hybrids between the subspecies.

### ﻿Diversity of *Ae.comosa* in localization of rDNA clusters

**Figure 4.**
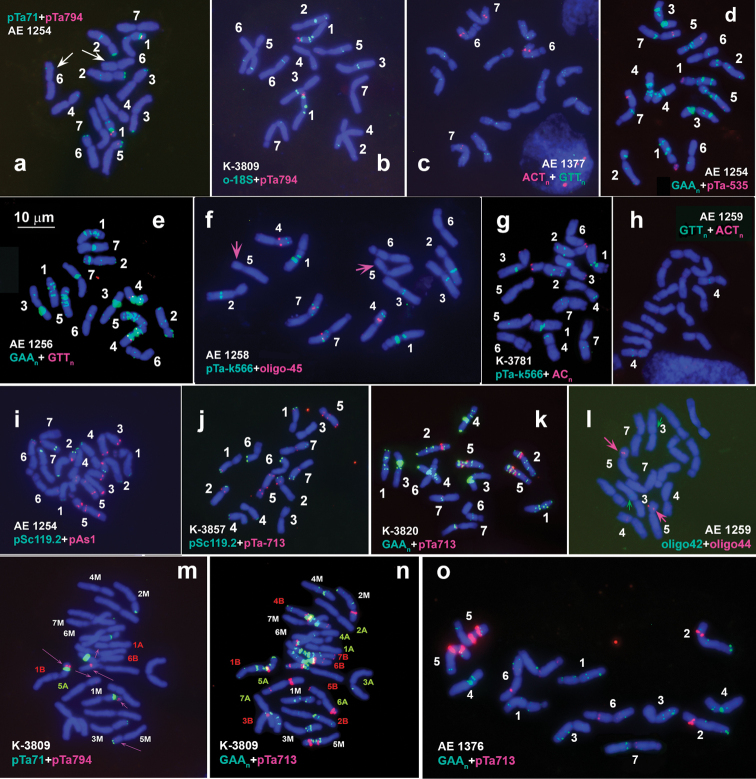
Localization of repeated DNA sequences on metaphase chromosomes of Ae.comosasubsp.comosa. Accession numbers and probe names are shwon on each plate; probe color corresponds to signal color. Chromosomes are designated with numerals according to genetic groups. Scale bar: 10 µm.

**Figure 5.**
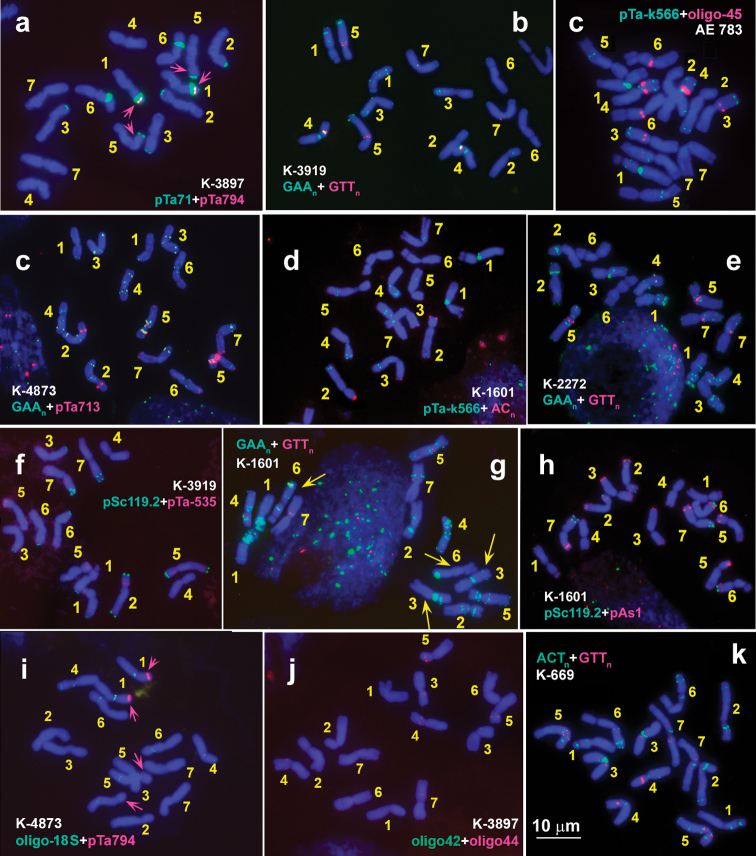
Localization of repeated DNA sequences on metaphase chromosomes of Ae.comosasubsp.heldreichii. Accession numbers and probe names are shown on each plate; probe color corresponds to signal color. Chromosomes are designated with numerals according to genetic groups. Scale bar: 10 µm.

Clusters of rDNA were mapped on chromosomes of 36 *Ae.comosa* accessions by FISH with probes oligo-pTa71-2 (thereafter pTa71), oligo-pTa-794 (5S rDNA), and oligo-18S (thereafter o-18S). Comparion of labeling patterns obtained using pTa71 and o-18S probes reveals intrinstic feature. The pTa71 visualizes all minor and major rDNA loci (Figs 4a, 5a), while o-18S fails to reveal major NORs and *Ae.comosa*-specific minor NORs located terminally on 2MS, 3MS and 5MS arms (Figs 4b, 5i). By contrast, o-18S is more efficient in detecting minor NORs located in interstitial and pericentromeric regions of many M-genome chromosomes (Figs 4b, 5i; Suppl. material 1: fig. S1c16, h04–h06, h10).

**Figure 6.**
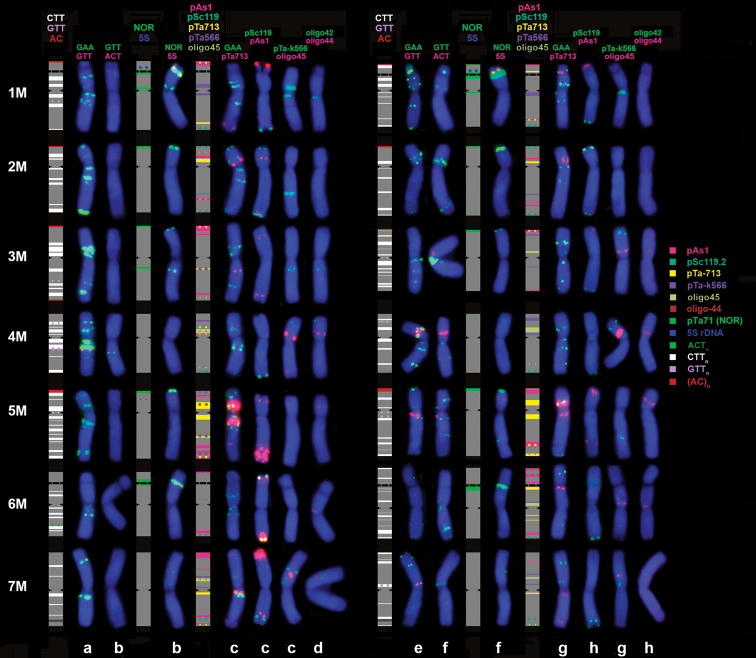
Idiograms and chromosomal images showing the distribution of repetitive DNA families on chromosomes of *comosa* (left side) and *heldreichii* (right side). Probe names are shown on the top of the figure; probe color corresponds to signal color. Accession codes are given in the bottom: **a** AE 1257 **b** AE 1259 **c** AE 1258 **d** AE 1377 **e** AE 117 **f** K-669 **g** AE 783 **h** K-3897.

In karyotype of *Ae.comosa* major NORs are located on chromosomes 1M and 6M (Fig. 6). Signals on 1M and 6M detected with pTa71 probe are usually equal in size, however in AE 1254 (*comosa*) and K-3806 (*heldreichii*) the signals on 6M are significantly smaller compared to 1M (Fig. 4a, Suppl. material 1: fig. S1, c06, h03). As mentioned above, these accessions carry only one pair of SAT chromosomes; thus, inactivation of NORs on 6M is associated with/ or caused by elimination of the 45S rRNA gene sequences from the respective loci.

The pTa71 probe produces distinct signals in subtelomeric regions of short arms of 2M, 3M, and 5M chromosomes (minor NORs) in all accessions of both *comosa* and *heldreichii*, however, these loci are not visualized by o-18S probe. The latter ribosomal probe however reveals weak signals in pericentromeric region of 1ML, distal region of 2ML and 4MS, and two minor loci in the proximal half of 7M short arm; all these sites are common for both subspecies. By contrast, several sites discriminate *comosa* from *heldreichii* (Fig. 6, Suppl. material 1: fig. S1). These are: (1) the locus of variable intensity in a proximal part of chromosome 3M is observed in nearly all (with one exception) *comosa* genotypes, but not in *heldreichii*; (2) most *comosa* carry two minor pericentromeric o-18S sites in the opposite arms of chromosome 1M, while *heldreichi* possesses signal only in the long arm; (3) minor pericentromeric rDNA site on chromosome 4M is usually located in the long arm of *comosa*, but in the short arm of 4M^h^ of *heldreichii*; (4) most *comosa* accessions possess two weak intercalary pTa71 sites in the long arm of 2M, and only one distal site is present in *heldreichii*.

Two pairs of 5S rDNA loci with unequal size are revealed in all *Ae.comosa* accessions. The signal on 1M is much larger than that on 5M and arrears distally to NOR. The signal on 5M is very faint, especially in *heldreichii*, and occurs in the middle of short arm (Fig. 6).

Based on hybridization pattern of 5S and 45S rDNA probes we identify a reciprocal translocation between 1M and 6M chromosomes in K-3308 (Suppl. material 1: fig. S1c13), which caused the alterations in morphology of the SAT chromosomes observed in all Group 3 accessions.

### ﻿Diversity of *Ae.comosa* in the distribution of microsatellite probes

Four microsatellite probes: GAA_10_, GTT_10_, ACT_10_, and AC_20_ were mapped on chromosomes of *Ae.comosa*. Hybridization with GAA_10_ (Figs 4d, e, k, 5b, c, e, g) results in patterns similar to C-banding (C-banding-like patterns). Thus, *comosa* chromosomes carry numerous GAA_n_ signals of variable size in subterminal, interstitial and pericentromeric chromosome regions. Compared to *comosa*, *heldreichii* possess lesser number of (GAA)_n_ sites, most of which with low or moderate intensity. Hybridization signals in *heldreichii* appear predominantly in subtelomeric and pericentromeric chromosome regions, rarer interstitially (Fig. 6).

Giant GAA_n_ signals exceeding the respective sites on 1M of *comosa* are detected on the short arm of 1M^h^ of few *heldreichii* accessions. Both *comosa* and *heldreichii* exhibit an extremely broad polymorphism of GAA_n_-labeling patterns (Suppl. material 2: fig. S2, Suppl. material 3: fig. S3); differences between accessions, between individual plants within the accession (Suppl. material 3: fig. S3h–i, t, u, Suppl. material 4: fig. S4d–f, k, l) and even between homologous chromosomes (heteromorphism of homologues) have frequently been observed (Fig. 5g; Suppl. material 3: fig. S3e). Using the GAA_n_ probe we confirm the 1M-6M translocation in K-3308 (*comosa*) and identify a similar translocation in K-4873 (*heldreichii*) accession (Suppl. material 2: fig. S2d, e, Suppl. material 3: fig. S3j).

Most obvious differences between the subspecies show chromosomes 3M and 3M^h^ (Fig. 6), which differ even in size and morphology. 3M of *comosa* is larger and more asymmetric (submetacentric) than 3M^h^ of *heldreichii* (metacentric), due to the loss of a distal part of the long arm. The short arm of 3M (*comosa*) carries two prominent, often fused GAA_n_ clusters in a proximal part and several smaller sites in the distal and, rarer, in the proximal third of the long arm. A slightly deviant hybridization pattern of 3M in K-3857 (*comosa*) can be caused by large pericentric inversion (Fig. 4q). Chromosome 3M^h^ of *heldreichii* shows highly polymorphic labeling patterns (Suppl. material 3: fig. S3), however three intercalary GAA_n_-sites in the long arm are constantly present. Most proximal GAA_n_ site varies in signal intensity from huge (K-669, K-1601, K-2272, K-2432) to medium or even small (K-3914, K-3919).

Among all *comosa* accessions, K-3857, AE 1376 and AE 1377 from Greece exhibit most deviant GAA_n_ patterns (Suppl. material 2: fig. S2n, p, q). According to karyotype analysis, K-1601 is highly heterogeneous and consists of genotypes, belonging to *comosa* and *heldreichii* types, and some seedling prove to be hybrids, including hybrids between the subspecies (Suppl. material 3: fig. S3e). Hybrids between the subspecies and even with an unknown tetraploid wheat species have been identified within the accession K-3809 (Fig. 3m, n).

Distribution of GTT_10_ probe discriminates subspecies comosa from *heldreichii*. Two small, but sharp intercalary signals appear in short and long arms of 4M in all *comosa* accessions (Fig. 4e). These GTT_n_ sites are located at the borders of large pericentromeric GAA_n_ complex (Suppl. material 2: fig. S2g). All studied *heldreichii* accessions possess distinct GTT_n_ signals on three chromosome pairs, 4M (middle of short arm), 5M and 7M (pericentromeric regions) (Fig. 5b, e, g; Suppl. material 3: fig. S3a). In addition, very weak, fuzzy hybridization signals of GTT_n_ probe may appear in regions overlapping with GAA_n_ in both *comosa* and *heldreichii* accessions.

Poor hybridization with ACT_10_ probe is observed in eight *Ae.comosa* accessions examined in our study; chromosomal regions corresponding to C-bands/ GAA_n_ sites show a little brighter intensity (Figs 4c, h, 5k). Sharp signals appear on chromosomes 6ML and 7ML of only one *comosa* accession – AE 1377 (Fig. 4c).

Hybridization of (AC)_20_ repeat on chromosomes of *comosa* and *heldreichii* results in similar patterns: small subtelomeric signals appear on chromosome arms 1MS, 2MS, 3MS/ 3ML, and 5MS (Suppl. material 4: fig. S4g, h, p). These are the sites in which minor subtelomeric NORs are also detected (Figs 4g, 5d; Suppl. material 1: fig. S1 c01, c03, h02).

### ﻿Polymorphism in the distribution of satellite DNAs: pTa-713 family

Most prominent pTa-713 clusters appear in the short arm of 2M and in very proximal parts of both arms of 5M. Two smaller sites are observed in subtelomeric and pericentromeric regions of the long arm of 7M of all studied accessions of *Ae.comosa* (Figs 4j, k, 5c, 6). Signals in a terminal part of 1MS, a distal part of 1ML, and in subtelomeric region of 5ML are detected in several accessions belonging to both subspecies, however, some polymorphic pTa-713 sites are specific for either *comosa* or *heldreichii* (Suppl. material 5: fig. S5, Suppl. material 6: fig. S6).

Thus, sixteen of 20 accessions of *comosa* carry small signal in a proximal third of 3ML, while the signal in the middle of 5ML is detected in more than a half *comosa* accessions (Suppl. material 5: fig. S5). AE 1376 and AE 1377 (*comosa*) show the unique pTa-713 patterns on 3M chromosome consisting of clear signal in the terminus of short arm and fuzzy signal in a distal part of the long arm (Suppl. material 5: fig. S5g, h, shown with pink arrows), which are not observed in *comosa* and *heldreichii* accessions, except K-3811 (*heldreichii*) (Suppl. material 6: fig. S6e). Most *comosa* also possess two faint signals in the short arm of 4M. Two *comosa*’s lack these marker sites, but carry a small signal in the distal part of 4ML arm (Suppl. material 5: fig. S5o, t). Many small sites in unusual positions appear in Turkish accession K-3787 (*comosa*).

All *heldreichii* accessions show characteristic labeling pattern of chromosome 6M^h^, which carries clear pTa713 signal in the short arm, either adjacent to secondary constriction (8 of 13 accessions) or in the middle of satellite (2 accessions), and two signals in the proximal half of the long arm (Suppl. material 6: fig. S6). We consider such pattern as *heldreichii*-specific, although it is not observed in two “*comosa*-like” genotypes of K-1601. These genotypes carry “short” satellite on 6M^h^, similar to chromosome 6M of *comosa*. A unique prominent pTa-713 site is detected in a distal part of 5M^h^L of K-3914 (*heldreichii*) (Suppl. material 6: fig. S6a).

### ﻿Polymorphism in the distribution of satellite DNAs: pSc119.2 family

Signals of pSc119.2 probe appear in subtelomeric regions of 1ML, 2MS+2ML, and 6ML arms and in distal (*comosa*) or terminal + distal parts of 7ML (mainly *heldreichii*) of all studied accessions of *Ae.comosa* (Figs 4i, j, 5f, h). The pSc119.2 signals in the distal part of 4MS and a terminus of 4ML occur more frequently in *comosa* (Fig. 6; Suppl. material 7: fig. S7). No pSc119.2 signals appear on 5M chromosome of 26 studied accessions of *Ae.comosa* belonging to both subspecies, and only three accessions (all *heldreichii*) carry small pSc119.2 site in the terminus of 3M^h^L (Suppl. material 7: fig. S7o, r).

### ﻿Polymorphism in the distribution of satellite DNAs: pAs1 and pTa-535 families

Comparison of labeling patterns obtained using pAs1 and pTa-535 probes (e.g., Fig. 4d, I, red color) shows that all pTa-535 sites overlap with pAs1 sites, however many pAs1 sites are not detected by pTa-535 (Suppl. material 3: fig. S3t). Based on this observation, we choose pAs1 for polymorphism assessment in *Ae.comosa*. In general, *comosa* exhibits more intense hybridization with pAs1, however broad variation of labeling patterns observed in both subspecies preclude their discrimination using this probe (Suppl. material 7: fig. S7). Prominent pAs1 clusters occur in terminus of the SAT of 1M chromosome of AE 115, AE 1256, AE 1258, AE 1378, K-3309, K-3787, K-3819, K-3820 (*comosa*, see Suppl. material 7: fig. S7a, c, h, i) and K-2432 (*heldreichii*, see Suppl. material 7: fig. S7l) accessions. Accessions AE 1256, AE 1259, AE 1378, K-3787, K-3819, K-3820, K-3920, and K-3857 contain large signals in subtelomeric region of 3MS (Suppl. material 7: fig. S7c, f, i), K-3820 and AE 1258 – in subterminal part of 6ML, and accessions AE 1258, AE 1259, K-3309, K-3787 (*comosa*) and K-1601 (*heldreichii*) – in subtelomeric region of the 7M short arm (Suppl. material 7: fig. S7e, f, h). Prominent interstitial pAs1 clusters emerge in a distal part of 5M long arm of AE1254 and AE1258 (*comosa*), and K-3914 (*heldreichii*). All chromosomes except 4M possess medium to small hybridization sites located in interstitial and more frequently in distal and subtelomeric chromosome regions. Hybridization with pSc119.2 and pAs1 probes confirms the translocation 1M-6M in *comosa* accession K-3309 (Suppl. material 7: fig. S7h).

### ﻿Polymorphism in the distribution of satellite DNAs: pTa-k566 and oligo-45 families

The pTa-k566 sequence hybridizes to all chromosomes of *Ae.comosa* and labeling patterns varied between the accessions. Polymorphism observed on 1M, 2M, 3M, 4M, 6M, and 7M chromosomes is found to be subspecies-specific (Fig. 6, Suppl. material 4: fig. S4). Chromosomes 7M of *comosa* and *heldreichii* with similar probe distribution differ only in signal intensities, whereas other chromosomes carry pTa-k566 sites specific for only one of the subspecies. Chromosome 1M of *comosa* contains pTa-k566 sites at the both sides of the centromere, but only one site occurs on 1M^h^L of *heldreichii*. Median and distal pTa-k566 signals are present in the long arm of 2M of *comosa*, while only distal of them appears in *heldreichii*. Chromosome 3M of *comosa* carries prominent pTa-k566 site in the proximal region of the long arm, while the respective site on 3M^h^ of *heldreichii* is much weaker and the chromosome possess the second site distally in the short arm. Chromosome 4M of *comosa* lacks small proximal pTa-k566 site in the short arm, which occurs on 4M^h^ of all *heldreichii* accessions. *comosa* differs from *heldreichii* in the presence of clear pTa-k566 site in the proximal regions of 6M short arm, although it is also detected in two of 11 studied *comosa* accessions, namely K-3787 and AE 1377 (Suppl. material 4: fig. S4). Noteworthy that all pTa-k566 sites overlap with o-18S loci.

Chromosomes 1M and 2M do not possess signals of oligo-45 probe. Labeling patters of oligo-45 on chromosomes 4M and 7M of both *Ae.comosa* subspecies are similar, but signal intensity on 7MS is stronger in *comosa*. Some oligo-45 sites appear to be subspecies-specific. Thus, small interstitial signal in a distal part of 5MS is present only in *comosa*, whereas chromosomes 3M^h^ and 6M^h^ of *heldreichii* carry distinct oligo-45 sites proximally in the short arm (Suppl. material 4: fig. S4). Accessions K-4873 (*heldreichii*) is heterozygous in labeling pattern of oligo-45 on 5M^h^ chromosome, whereas AE 1377 (*comosa*) – of 6M chromosome (Suppl. material 4: fig. S4b, j). Besides this, accession AE 1377, being classified as *comosa*, carries variants of 1M, 2M, 3M, and 4M chromosomes typical for *heldreichii*, while 5M and 7M – typical for *comosa* (Suppl. material 4: fig. S4b).

### ﻿Polymorphism in the distribution of satellite DNAs: oligo-42 and oligo-44 families

No signals of oligo-42 probe are detected on chromosomes of five *heldreichii* accessions, and very weak, inconsistent signals are found on chromosomes of very few *comosa* accessions. Most frequently, signals occur in the long arm of 3M (Fig. 4l), but in AE 1377 oligo-42 signals are also observed on other chromosomes (Fig. 6). Positions of these sites coincide with the signals of o-18S probe. Owing to weakness and inconsistency of oligo-42 signals, this probe is considered non-informative for FISH-analysis of *Ae.comosa*.

All *Ae.comosa* accessions contain clear oligo-44 site approximately in the middle of 5M short arm (Figs 4l, 5j; Suppl. material 4: fig. S4). Additional, weaker signals emerge in the proximal part of 4M short arm and in a proximal third of 7M short arm. Besides them, many, but not all *heldreichii* accessions carry minor oligo-44 signals proximally in 3M short arm and in pericentromeric region of 6M short arm. Both *heldreichii*-specific sites are recorded in AE 1377 (*comosa*), which however exhibits also several *comosa*-specific karyotype features.

### ﻿Polymorphisms of electrophoretic patterns of gliadins

**Figure 7.**
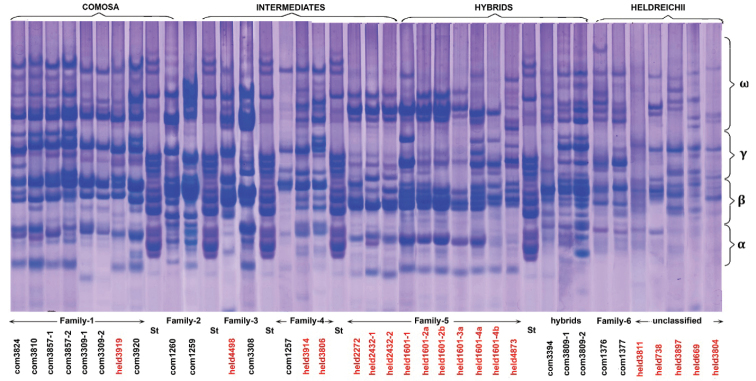
Electrophoretic spectra of Ae.comosasubsp.comosa (black) and subsp. heldreichii (red) accessions and their subdivision on families. St – the etalon spectra of wheat Bezostaya-1.

Genetic variability of *Ae.comosa* was also assessed using electrophoretic analysis of gliadins in 13 *comosa* and 13 *heldreichii* accessions. Comparison of gliadin profiles of all 26 accessions reveals an extremely broad intraspecific variation of *Ae.comosa*: each accession shows the unique profile. Spectra of *comosa* accessions are usually more “enriched in components” compared to *heldreichii* accessions (Fig. 7). This trend is manifested in a larger number of protein bands and their higher intensities, which is especially clear in the a-zone of electrophoretic spectra controlled by chromosomes of homoeologous group 6. From the other side, this trend is not mandatory because gliadin profiles of some *comosa* accessions (e.g., AE 1257, AE 1376, AE 1377) are very poor, whereas several *heldreichii* accessions exhibit rich spectra (K-3919; K-4498, some genotypes of K-1601).

**Figure 8.**
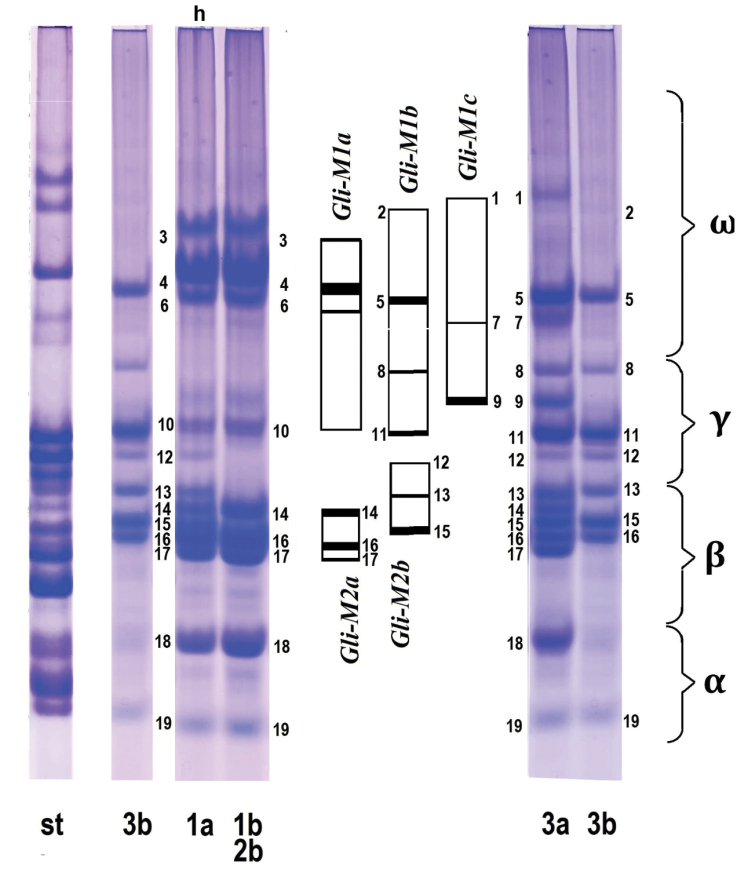
Diversity of the gliadin spectra detected in different grains taken from three individual spikes (1a and 1b; 2a and 2b, 3a and 3b) of *heldreichii* accession K-1601. St – an etalon wheat cultivar Bezostaya-1. Square blocks in the middle of the figure specify the blocks of gliadin components identified in eight genotypes of K-1601 (*Gli-M1a, Gli-M1b, Gli-1c; Gli-M2a, Gli-M2b*).

Several accessions analyzed in this study are genetically homogeneous and consist of genotypes with identical spectra (e.g., K-3914, K-3309, K-3920, AE 1376). Other accessions are found to be heterogeneous and show two or even more gliadin profiles (K-3857, K-3809 (*comosa*), K-1601 and K-2432 (*heldreichii*)). The broadest variation of gliadin patterns is detected in K-1601. We identified six variants of electrophoretic spectra among eight individual grains taken from four spikes; they differ in position of polypeptide bands in γ- and ω-zones (Fig. 8). The spectra 1a and 1b are more typical for *comosa*, whereas 2a and 3b (different spikes) – for *heldreichii*. The spectrum 3a contains bands present on 2a and 3b and probably represents a hybrid between these two genotypes. Similarly, the spectrum 1a may correspond to hybrid between 1b and 3b (Fig. 8). Another heterogeneous accession – K-3809 (*comosa*) is characterized by spectra highly enriched with gliadin components, which differ significantly from other *Ae.comosa* in the number, position and size of protein bands. This genotype can represent a hybrid of *Ae.comosa* with unknown 4× wheat species.

Comparison of gliadin patterns of different accessions of *Ae.comosa* shows that several combinations of polypeptide bands on electrophoretic spectra always appear together and are inherited as blocks of components (Fig. 7). Such coincidence means that (1) these bands are controlled by a common locus, which, in turn, indicates (2) that accessions under comparison are genetically related. Based on the presence of such common blocks we discriminate six gliadin Families among 26 *Ae.comosa* accessions (Fig. 7): (1): K-3824; K-3309, K-3810, K-3824; K-3857, K-3920 – *comosa*, K-3919 – *heldreichii*; (2): AE 1259, AE 1260 – *comosa*; (3): K-3308 – *comosa*, K-4498 – *heldreichii*; (4): AE 1257 – *comosa*, K-3806, K-3914 – *heldreichii*; (5): K-1601, K-2272, K-2432, K-4873 – *heldreichii*; (6): AE-1377, AE-1376 – *comosa*.

Families include accessions mainly from one subspecies. Thus, Families 1 and 2, which exhibited the richest spectra, consist of predominantly *comosa* accessions. Families 4 and 6 and partially Family 5 having relatively poor spectra are mainly composed by *heldrechii* accessions. Although both accessions from Family 6 have been assigned to subspecies comosa, their gliadin spectra share more common features with the spectra of *heldrechii* accessions AE 783, K-669, K-3804, K-3811, and K-3897 than with those of *comosa*. Each of the five abovementioned *heldrechii* accessions show unique gliadin profile, which cannot be assigned to either one of the families due to dissimilarities in position and intensity of polypeptide bands on electrophoretic spectra. High ratio of *heldrechii* accessions with the unique gliadin spectra is an indicative of higher variability of this subspecies compared to subspecies comosa.

## ﻿Discussion

Our current results and the data available from literature (Song et al. 2020) demonstrate that the levels of intraspecific diversity in *Ae.comosa* significantly vary depending on markers used for their assessment. Thus, FISH with GAA_n_ probe and gliadin electrophoresis uncover an extremely broad polymorphism of this species. High effectiveness of these markers for the analysis of intra- and interspecific diversity, evaluation of population structure, for characterization of individual genotypes has been proved in many publications (Metakovsky et al. 1989; Badaeva et al. 1990, 2015b, 2022; Masci et al. 1992; Pomortsev et al. 2011; Keskin et al. 2015; Jiang et al. 2017; Majka et al. 2017; Song et al. 2020). We find that, from one side, distribution of GAA_n_ sites is species- and chromosomes specific allowing identification of all individual chromosomes. From the other side, each accession carries a unique combination of polymorphic GAA_n_ sites and unique gliadin spectrum comprising their individuality.

Comparison of the GAA_n_ patterns of *Ae.comosa* chromosomes obtained in a current study and those reported previously (Molnár et al. 2016; Song et al. 2020) with the C-banding patterns (Teoh et al. 1983; Friebe et al. 1996; Badaeva et al. 1999) show that positions of GAA_n_ overlap with location of C-bands. Two other microsatellite sequences – GTT_n_ and ACT_n_, which are known to be the components of constitutive heterochromatin in chromosomes of wheat and other grass species (Pedersen and Langridge 1997; Cuadrado et al. 2000; Cuadrado et al. 2008a, b; Luo et al. 2017, 2018; Zhang et al. 2022) are detected in minor quantities. The (AC)_n_ sites co-localize with positions of minor NORs visualized with pTa71 probe on 1MS, 2MS, 3MS/3ML, and 5MS arms. These facts indicate that heterochromatin regions detected using Giemsa C-banding on *Ae.comosa* chromosomes are composed by predominantly GAA_n_ repeat, which is typical for the Triticeae, except diploid *Thinopyrum* Á.Löve, 1980 einkorn wheat and *Ae.tauschii*. Chromosomes of these species contain only little amounts of microsatellite sequences or do not possess them at all (Badaeva et al. 2015a, 2019a, b; Linc et al. 2017; Li et al. 2018; Zhao et al. 2018; Ebrahimzadegan et al. 2021).

Although labeling patterns of GAA_n_ probe prove to be highly informative for *Ae.comosa* chromosome identification and authentication of gene bank accessions, they are too polymorphic and complicated for broad-scale phylogenetic analyses. A similar complexity and ambiguity is found for gliadin profiles. The appropriate markers should be relatively simple and easy to score and should generate specific and reproducible patterns. Eight out of 15 FISH probes used in our study fit these criteria: the 5S and 45S rDNAs, pAs1, pSc119.2, pTa-713, pTa-k566, oligo-44, and oligo-45 probes (oligo-42 and ACT_n_ are found to be low informative for the analysis of *Ae.comosa* chromosomes due to weak and inconsistent labeling patterns). We used these eight probes for verification of the M-genome chromosome classification and for the assessment of intraspecific diversity of *Ae.comosa*.

Our study reveals an interesting feature of two oligo-probes designed for the detection of 45S rDNA loci. The oligo-pTa71-2 probe developed by Tang et al. (2014) is homologous to wheat rDNA 25S-18S intergenic region *Eco*RI-*Bam*HI fragment (X-7841.1). This oligo-probe proves to be effective for the analysis of wheat and *Aegilops* species, however it fails to detect NORs in most other plant taxa, including, for example, barley, oat, or *Erantus* (Ranunculaceae) (Mitrenina et al. 2021). Owing to this, we tried to design a new oligo-probe for rDNA loci (based on genome sequence of *Aegilopstauschii*), which will be applicable for many plant species. Nucleotide sequence of a newly designed oligo-18S is homologous to highly conservative region of 18S rDNA gene of *Aegilops*, *Triticum*, *Hordeum*, *Musa*, and *Iris* (Suppl. material 8: fig. S8d) and it was able to detect major NORs on barley chromosomes, although signals were very weak. The o-18S hybridizes to chromosomes of many *Aegilops* species, however it was more effective for the detection of minor NORs (Suppl. material 8: fig. S8c). In contrast to pTa71 probe obtained from plasmid DNA or oligo-pTa71, o-18S does not detect major NORs and fails to reveal marker terminal 45S rDNA loci on 2M, 3M and 5M chromosomes (Suppl. material 8: fig. S8a, b). Currently we have no explanation of this phenomenon.

Correct chromosome classification is essential for phylogenetic analyses. Nomenclatures suggested for *Ae.comosa* chromosome classification have been built on different principles. In early studies, the authors followed the rules of cytological nomenclature: chromosomes are arranged according to decreasing length and arm ratio (Chennaveeraiah 1960; Teoh et al. 1983; Georgiou et al. 1992). Currently most chromosome classifications of the Triticeae species are based on homoeology with common wheat chromosomes – genetic nomenclature. Although genetic nomenclature of *Ae.comosa* chromosomes have been suggested in several publications (Badaeva et al. 1996a; Friebe et al. 1996; Molnár et al. 2011, 2016; Liu et al. 2019; Song et al. 2020; Said et al. 2021), classification of some chromosomes is still controversial. Two chromosomes – 2M and 5M, prove to be most difficult for discrimination owing to same DNA content, similar morphology, pAs1 labeling patterns, heterochromatin content and distribution.

**Figure 9.**
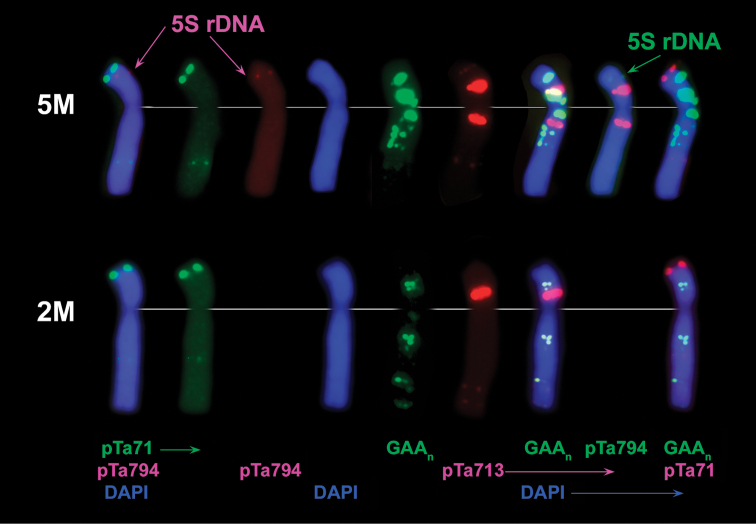
Characterization of 2M and 5M chromosomes of *Ae.comosa* using sequential FISH with (round 1) pTa794 (5S rDNA), pTa71 (45S rDNA), followed by (round 2) GAA_10_ and pTa-713 probes. Probe names are shown on the bottom, probe color corresponds to signal color. Arrows point to 5S rDNA loci on chromosome 5M.

Chromosome 2M was first identified and assigned to genetic group 2 by S. Nasuda et al. (1998) using RFLP, GISH and C-banding techniques in *Ae.comosa*, wheat cultivar Compair, and wheat-*Ae.comosa* 2A/ 2M and 2D/ 2M translocation lines. Classification of 2M and 5M was further validated by M. Said et al. (2021) based on FISH mapping of tandem repeats and wheat single-gene probes. In a current study we confirmed classification of 2M and 5M by using DNA probes pTa794 (5S rDNA) and oligo-44. This is because the 5S rDNA loci in *Triticum* Linneaus, 1753 and *Aegilops* occur only on group 1 and 5 chromosomes (Dvořák et al. 1989), and in diploid *Aegilops* species the respective signals appear in a distal part of group 1 chromosomes and in the middle of short arm of group 5 chromosomes (Badaeva et al. 1996b). The 5S rDNA signal on 5M localizes in the middle of short arm, thus this chromosome belongs to genetic group 5. No signals of 5S rDNA probe are detected on another chromosome, which is designated 2M (Fig. 9).

Another validation of genetic group for chromosome 5M come from hybridization pattern of oligo-44 probe. This probe was developed from chromosome-specific tandem repeats of wheat (Tang et al. 2018b) and mapped on all group 5 chromosomes, 3A and 7A of Chinese Spring. A little later, T. Lang at al. (2019) using bioinformatics tools identified the homologous minisatellite repeat, Ta-3A1 from the wheat genome assembly and localized it on group 5 chromosomes of wheat (2x, 4x and 6x), rye, *Aegilops*, *Dasypyrum* (Cosson et Durieu) T.Durand, 1888 and *Thinopyrum* species. The CL241 probe, another homolog of oligo-44 isolated from *Ae.crassa* genome, was also mapped to all group 5 chromosomes of *Ae.tauschii* (2n=2x=14, DD), tetraploid and hexaploid *Ae.crassa* (2n=4x=28, D^1^D^1^X^cr^X^cr^ and 2n=6x=42, D^1^D^1^X^cr^X^cr^D^2^D^2^ (Kroupin et al. 2022).

Interestingly, that in *Ae.comosa* we reveal minor oligo-44 signals on chromosomes 3M and 7M, the homoeologs of 3A and 7A, which also possess the TA-3A1 sites in karyotypes of diploid, tetraploid and hexaploid wheat species, but not in *Aegilops* (Lang et al. 2019).

Another interesting outcome from our study is a possibility of discrimination of the two *Ae.comosa* subspecies using chromosomal markers. From one side, all thirty-six accessions of *Ae.comosa* included in the analyses show similar karyotype structures, distribution of rDNA loci, labeling patterns of repetitive DNA sequences indicating that they all belong to one biological species. Despite these similarities, we found clear and reproducible differences between the subspecies in morphology, C-banding and FISH patterns of two chromosome pairs – 3M and 6M. We hypothesize that these two chromosomes were involved in the subspecies-specific translocation identified earlier in *Ae.comosa* by mean of meiotic analysis (Kihara 1940). This translocation probably led to the shortage of the long arm of 3M and the increase in the satellite length on 6M of *heldreichii* compared to *comosa*.

Interestingly, all *heldreichii* accessions carrying large SAT on chromosome 6M showed relatively poor gliadin profiles (Fig. 8). Considering these facts, we suggest that the 3M-6M translocation can change the functioning of gliadin loci on 6M^h^ chromosome resulting in depletion of gliadin spectra in the α-zone. Intraspecific differentiation of *Ae.comosa* (Groups I and II) in the morphology of SAT chromosomes observed in our study and reported previously (Karataglis 1975) is caused by this subspecies-specific translocation, whereas the Group III probably evolved due to reciprocal 1M-6M translocation identified here in *comosa* K-3308, K-3309, K-3787 (all from Turkey) and K-4873 (*heldreichii*) accessions using FISH with rDNA, pTa-713, and GAA_n_ probes.

Peculiarities of C-banding (Teoh et al. 1983; Friebe et al. 1996; Badaeva et al. 1999)/ GAA_n_ patterns also allow discrimination of *Ae.comosa* subspecies indicating that their divergence was accompanied by amplification/ elimination and re-distribution of microsatellite repeats. These results however contradict the observations of Z. Song et al. (2020), who have not revealed any differences between the subspecies in GAA_n_ labeling patterns. These authors, however, did not provide complete karyotypes of accessions used in their analyses, which does not permit the direct comparison of our data.

Differences between the subspecies are most clearly detected using a combination of pTa-k566 and oligo-45 probes; the pTa-713 also shows subspecies-specific patterns. These three probes prove to be best choice for the precise discrimination of *comosa* from *heldreichii* accessions using FISH markers. Most *Ae.comosa* chromosomes show rather conservative patterns, while diagnostic sites appear mainly on 3M or 6M. Different repeats are often accumulated in a single cluster. Such complex cluster, composed of 45S rDNA, pTa-713, pTa-k566, oligo-42, and, rarely, GAA_n_ appears in a proximal third of the long arm of 3M (*comosa*) (Fig. 6). These repeats are mainly absent or found in minor quantities in the long arm of 3M^h^ (*heldreichii*), which contains small cluster consisting of oligo-42 and oligo-45 in the short arm. Clear signals of pTa-713, oligo-44 and oligo-45 probes are detected on chromosome 6M^h^ of *heldreichii*, but they are lacking in 6M of *Ae.comosa*. Although pAs1 probe cannot reliably discriminate *comosa* from *heldreichii*, pSc119.2-labeling pattern of 7M shows differences between the subspecies, although with few exceptions. Thus, two *comosa* accessions – AE 1377 and K-3809, carry two pSc119.2 sites on 7ML, which is typical for *heldreichii*. These accessions however are deviant and share karyotypic features of both subspecies. Cytogenetic analysis reveals that some grains of K-3809 represent hybrids of *Ae.comosa* with unknown tetraploid wheat, which can explain a deviant gliadin profile of this accession, and one genotype possessing heteromorphic chromosome 5M is probably a derivative of *comosa*×heldreichii cross.

The significant role of hybridization in evolution and diversification of *Ae.comosa* is supported by other studies (Georgiou et al. 1992; Song et al. 2020). Thus, these papers described many heterozygotes in *Ae.comosa*, and in a current study we found genotypes segregating in labeling patterns of one to all seven chromosome pairs (see Suppl. material 1: fig. S1, h10, Suppl. material 3: fig. S3e, Suppl. material 4: fig. S4j), which point to their hybrid origin. Accession K-1601 (*heldreichii*) shows the highest heterogeneity: each of the seven genotypes examined by FISH and 5/8 genotypes analyzed by gliadin electrophoresis show unique patterns. Karyotypic features of some K-1601 genotypes correspond to *heldreichii* subspecies, while other share similarities with *comosa*. A similar trend is uncovered by gliadin analysis. Both FISH and gliadin electrophoresis identify many heterozygotes in K-1601, which may represent recent hybrids, including hybrids between the subspecies. It should be mentioned, however, that no variation in spike morphology (all *heldreichii*-like, see Fig. 2) has been identified between individual plants of this accession.

Three accessions, AE 1376, AE 1377 and K-3857 assigned to subspecies comosa based on botanical characters, combine chromosomal features of both *Ae.comosa* subspecies assuming that they might have hybrid origin. Gliadin analysis supports closer relations of AE 1376 and AE 1377 with *heldreichii* than with *comosa* indicating that taxonomical position of these accessions should be verified. Probably these forms emerged via hybridization of *comosa* and *heldreichii* followed by karyotype stabilization toward *heldreichii* (AE 1376, AE 1377) or *comosa* (K-3857) parent. In contrast to K-1601 or K-3809, these three accessions are cytogenetically stable and genetically uniform. Most likely, they emerged via *comosa*×heldreichii hybridization long time ago, and hybrid forms become stabilized over generations. Based on these facts we suggest that hybridization, including hybridization between subspecies, plays an important role in broadening genome diversity of this grass. It can be facilitated by following factors:

- *comosa* and *heldreichii* often grow together in mix stands (Zhukovsky 1928; Van Slageren 1994);
- heading and flowering time of *comosa* and *heldreichii* overlap (Boguslavsky and Golik 2003);
- *Ae.comosa* is considered as autogamous species, however, open pollination could be more common event than it is usually believed (Georgiou et al. 1992; Boguslavsky and Golik 2003);
- hybrids between the subspecies are partially fertile (Kihara 1940).

Summarizing results of a current study, we recommend the following set of markers for the precise identification of individual chromosomes and for discrimination of *Ae.comosa* subspecies using FISH markers (Table 2).

**Table 2.** Probe combinations for the M-genome chromosome identification and discrimination of *Ae.comosa* subspecies (according to Fig. 6).

Chr #	Markers common for subspecies	Markers discriminating subspecies
1M	Major NOR (pTa-71) in short arm;	Proximal o-18S/ pTa-k566 site in the short arm (*comosa*)
	5S rDNA locus in the satellite;	
	terminal (AC)_n_ site in satellite;	
	pSc119.2 site in long and pAs1/ pTa-535 site in short arm; proximal o-18S/ pTa-k566 site in long arm.	
2M	Minor NOR in short arm overlapping with (AC)_n_ site; pSc119.2 signals in both arms;	Intercalary o-18S/ pTa-k566 site in the middle of long arm (*comosa*).
	pAs1 signals in short and long arms;	
	distal o-18S/ pTa-k566 site in long arm;	
	large pTa-713 cluster in short arm.	
3M	Minor NOR in short arm overlapping with (AC)_n_ site; subterminal pAs1/pTa-535 cluster(s) of various intensity in short (and long) arm(s)	Metacentric (*heldreichii*) vs submetacentric (*comosa*);
		GAA_n_ patterns;
		cluster pTa71+pTa-713+pTa-k566+o-18S in long arm (*comosa*)/ cluster
		pTa-k566 in short arm (*heldreichii*);
		oligo-45 site in short arm (*heldreichii*).
4M	Minor distal NOR in short arm;	pTa71/ o-18S in short (*heldreichii*) vs long (*comosa*) arms,
	prominent oligo-45 cluster in short and small – in long arm;	
	Oligo-44 site overlapping with oligo-45;	proximal pTa-k566 site in short arm (*heldreichii*);
	One-two faint pTa-713 sites in short arm.	
		two faint (*comosa*) vs one clear (*heldreichii*) GTT_n_ sites in short arm
5M	Minor NOR in short arm overlapping with (AC)_n_;	pericentromeric GTT_n_ cluster (*heldreichii*);
	5S rDNA site in the middle of short arm;	
		oligo-45 site in short arm (*comosa*).
	Two prominent pTa-713 clusters;	
	pTa-k566 site in long arm;	
	oligo-44 site in short arm;	
	pSc119.2 signals are mainly absent;	
	pAs1 sites distally in the long arm and terminally in the short arm.	
6M	Satellite in physically longer arm carrying major NOR;	Medium (*comosa*) vs large (*heldreichii*) satellite;
	terminal pSc119.2 and distal pAs1 sites in the arm, opposite to NOR.	
		small pTa-713 sites in both arms (*heldreichii*);
		oligo-44, oligo-45, and pTa-k566 sites in the SAT arm (*heldreichii*).
7M	proximal pTa71/ o-18S/ pTa-k566 sites in short arm;	two (*heldreichii*) vs one (*comosa*) pSc119.2 sites in long arm;
	oligo-45 site in short arm;	
	pTa-713 sites in subtelomeric and proximal regions of long arm.	GTT_n_ cluster in proximal part of short arm (*heldreichii*);
		o-18S, pTa-k566, oligo-45 signal intensities

## ﻿Conclusions

FISH and gliadin electrophoresis reveal broad intraspecific polymorphism of GAA_n_ patterns and gliadin profiles of *Ae.comosa* allowing not only genetic authentication of gene bank accessions, but also discrimination between the subspecies. Application of these markers however will be too complicated for the broad-scale phylogenetic analyses.

By using group-specific FISH markers, we justify classification of 2M and 5M chromosomes of *Ae.comosa* and suggest a set of DNA probes for the precise identification of each of the seven M-genome chromosomes.

Two subspecies of *Ae.comosa* – *comosa* and *heldreichii*, are karyotypically distinct and diverge from each other as a result of subspecies-specific translocation 3M-6M, which probably affects functioning of gliadin locus. Divergence of subspecies was accompanied with amplification/ elimination and re-distribution of the repeated DNA sequences.

Three FISH probes, pTa-k566, pTa-713, and oligo-45 generate clear and reproducible patterns specific for *comosa* or *heldreichii* accessions; they can serve as reliable markers for discrimination of *Ae.comosa* subspecies.

An extremely broad genetic variability of GAA_n_-FISH patterns and gliadin profiles revealed in *Ae.comosa* – an endemic autogamous plant species (Van Slageren 1994), can be due to frequent occurrence of hybridization, including hybridization of *comosa* with *heldreichii* or with other neighboring wheat or *Aegilops* species.

## ﻿Author contributions

EB (Badaeva E.D.) planned and performed the experiments, analyzed data, wrote the first draft of the manuscript; KV: make chromosomal preparations and participates in FISH experiments; FA: carried gliadin electrophoresis, analyzed data; CN and BM: provide and characterized materials for this study; ZP: designed oligo probe; SS: synthesized oligo-probes; DA: analyzed gliadin spectra and wrote the manuscript. All authors read and approved the submitted version of the manuscript and agree to be personally accountable for their own contributions and for ensuring that questions related to the accuracy or integrity of any part of the work, even ones in which the author was not personally involved, are appropriately investigated, resolved, and documented in the literature.

## Supplementary materials

Supplementary material 1 Variation of hybridization patterns of pTa794 and o-18S or pTa71 rDNA probes Data type figure (TIF-file) Explanation note Variation of hybridization patterns of pTa794 (red) and o-18S (a, h, m, v–y) or pTa71 (b–g, i–l, n–u) rDNA probes (green) on chromosomes of following accessions of *Ae.comosa* (c01–c17 – subsp. comosa; h01–h14 – subsp. heldreichii): c01 – K-3819; c02 – K-3820; c03 – K-3781; c04 – K-3920; c05 – K-3810; c06 – AE 1254; c07 – AE 1257; c08 – AE 1258; c09 – AE 1259; c10 – AE 1376; c11 – AE 1377; c12 – AE 1378; c13 – K-3308; c14 – K-3787; c15 – K-3857; c16 – K-3809; c17 – K-3780; h01 – K-3804; h02 – K-1601; h03 – K-3806; h04 – K-2432; h05 – K-3911; h06 – K-3897; h07 – K-3919; h08 – K-4498; h09 – K-3914; h10 – K-3809; h11 – K-2272; h12 – AE 783; h13 – K-669; h14 – K-3824. Green arrows point to inactivated major NORs. Position of minor 45S rDNA loci specific for either *comosa* or *heldreichii* group are underlines with green lines. The 5S rDNA sites are shown with pink lines. Translocated 1M-6M chromosomes are arrowed. https://binary.pensoft.net/file/836913This dataset is made available under the Open Database License (http://opendatacommons.org/licenses/odbl/1.0/). The Open Database License (ODbL) is a license agreement intended to allow users to freely share, modify, and use this Dataset while maintaining this same freedom for others, provided that the original source and author(s) are credited.Ekaterina D. Badaeva, Violetta V. Kotseruba, Andnrey V. Fisenko, Nadezhda N. Chikida, Maria Kh. Belousova, Peter M. Zhurbenko, Sergei A. Surzhikov, Alexandra Yu. Dragovich

Supplementary material 2 Distribution of (GAA)_10_ microsatellite probe on chromosomes of different accessions of Ae.comosasubsp.comosa Data type figure (TIF-file) Explanation note Distribution of (GAA)_10_ microsatellite probe (green) on chromosomes of different accessions of Ae.comosasubsp.comosa: a – K-3810; b – K-3781; c – K-3787; d, e – K-3308; f – K-3309; g – AE 1256; h, i – AE 1254; j – AE 1257; k – AE 1258; l – AE 1259; m – AE 1378; n – K-3810; o – AE 1376; p – AE 1378; q – AE 1377; r – K-3857; s – K-3920; t – K-3820; u – K-3909; v, w – K-3809, x – K-3819. Translocated 1M-6M chromosomes are indicated. Position of (GTT)_n_ sites (red) on chromosome 4M of AE 1256 (g) is shown with red arrows. https://binary.pensoft.net/file/836914This dataset is made available under the Open Database License (http://opendatacommons.org/licenses/odbl/1.0/). The Open Database License (ODbL) is a license agreement intended to allow users to freely share, modify, and use this Dataset while maintaining this same freedom for others, provided that the original source and author(s) are credited.Ekaterina D. Badaeva, Violetta V. Kotseruba, Andnrey V. Fisenko, Nadezhda N. Chikida, Maria Kh. Belousova, Peter M. Zhurbenko, Sergei A. Surzhikov, Alexandra Yu. Dragovich

Supplementary material 3 Distribution of (GAA)_10_ microsatellite probe on chromosomes of different accessions of Ae.comosasubsp.heldreichii Data type figure (TIF-file) Explanation note Distribution of (GAA)_10_ microsatellite probe (green) on chromosomes of different accessions of Ae.comosasubsp.heldreichii: a – AE 117; b – K-3811; c – AE 783; d-f – K-1601; g – K-3919; h – K-3804; i – K-669; j – K-4873; k, l – K-2272; m – K-2432; n, o – K-3914; p – K-3897; q – K-4498. Translocated 1M^h^-6M^h^ chromosomes are indicated. Lane (e) presents karyotype of hybrid plant of K-1601, where “c” indicates homologous chromosomes of “*comosa*” type and “h” – homologous chromosome of *heldreichii* type. Localization of (GTT)_10_ probe (red) is shown with red arrows for accession AE 117 (a). https://binary.pensoft.net/file/836915This dataset is made available under the Open Database License (http://opendatacommons.org/licenses/odbl/1.0/). The Open Database License (ODbL) is a license agreement intended to allow users to freely share, modify, and use this Dataset while maintaining this same freedom for others, provided that the original source and author(s) are credited.Ekaterina D. Badaeva, Violetta V. Kotseruba, Andnrey V. Fisenko, Nadezhda N. Chikida, Maria Kh. Belousova, Peter M. Zhurbenko, Sergei A. Surzhikov, Alexandra Yu. Dragovich

Supplementary material 4 Distribution of oligo-42, oligo-44, oligo-45, (AC)_20_, and pTa-k566 probes on chromosomes of Ae.comosasubsp.comosa and subsp.heldreichii Data type figure (TIF-file) Explanation note Distribution of oligo-42, oligo-44, oligo-45, (AC)_20_, and pTa-k566 probes on chromosomes of Ae.comosasubsp.comosa (a-h) and subsp. heldreichii (i-p): a – K-3824; b – AE 1377; c – AE 1257; d – AE 1378; e – K-3820; f – AE 1258; g – K-3781; h – K-3819; i – K-3914; j – K-4873; k – K-3806; l – AE 783; m – K-3811; n – K-2432; o – K-669; p – K-1601. Probe names are shown on the top; probe color corresponds to signal color. Subspecies specific sites are underlined. Pink arrow points to heteromorphic signal. https://binary.pensoft.net/file/836916This dataset is made available under the Open Database License (http://opendatacommons.org/licenses/odbl/1.0/). The Open Database License (ODbL) is a license agreement intended to allow users to freely share, modify, and use this Dataset while maintaining this same freedom for others, provided that the original source and author(s) are credited.Ekaterina D. Badaeva, Violetta V. Kotseruba, Andnrey V. Fisenko, Nadezhda N. Chikida, Maria Kh. Belousova, Peter M. Zhurbenko, Sergei A. Surzhikov, Alexandra Yu. Dragovich

Supplementary material 5 Distribution of pTa-713 probe on chromosomes of different accessions of Ae.comosasubsp.comosa from Greece and Turkey Data type figure (TIF-file) Explanation note Distribution of pTa-713 probe (red) on chromosomes of different accessions of Ae.comosasubsp.comosa from Greece (a–o) and Turkey (p–t): a – AE 1258; b – AE 1254; c – AE 1256; d – AE 1257; e – AE 115; f – AE 1259; g – AE 1376; h – AE 1377; i – K-3810; j – K-3820; k – K-3819; l – AE 1378; m – K-3809; n – K-3808; o – K-3857; p – K-3309; q – K-3780; r – K-3781; s – K-3308; t – K-3787. Localization of (GAA)_10_ probe (green) is shown for accession AE 1258 (a). Positions of uncommon pTa-713 sites are arrowed. https://binary.pensoft.net/file/836917This dataset is made available under the Open Database License (http://opendatacommons.org/licenses/odbl/1.0/). The Open Database License (ODbL) is a license agreement intended to allow users to freely share, modify, and use this Dataset while maintaining this same freedom for others, provided that the original source and author(s) are credited.Ekaterina D. Badaeva, Violetta V. Kotseruba, Andnrey V. Fisenko, Nadezhda N. Chikida, Maria Kh. Belousova, Peter M. Zhurbenko, Sergei A. Surzhikov, Alexandra Yu. Dragovich

Supplementary material 6 Distribution of pTa-713 probe on chromosomes of different accessions of Ae.comosasubsp.heldreichii Data type figure (TIF-file) Explanation note Distribution of pTa-713 probe (red) on chromosomes of different accessions of Ae.comosasubsp.heldreichii: a – K-3414; b – K-3919; c – K-3897; d – K-1601; e – K-3811; f – K-2432; g – K-3804; h – K-4498; i – AE 783; j – K-4873; k – K-2272; l – AE 117; m –K-669. Translocated 1M^h^-6M^h^ chromosomes are indicated. Localization of (GAA)_10_ probe (green) is shown for accession K-3914 (a). https://binary.pensoft.net/file/836918This dataset is made available under the Open Database License (http://opendatacommons.org/licenses/odbl/1.0/). The Open Database License (ODbL) is a license agreement intended to allow users to freely share, modify, and use this Dataset while maintaining this same freedom for others, provided that the original source and author(s) are credited.Ekaterina D. Badaeva, Violetta V. Kotseruba, Andnrey V. Fisenko, Nadezhda N. Chikida, Maria Kh. Belousova, Peter M. Zhurbenko, Sergei A. Surzhikov, Alexandra Yu. Dragovich

Supplementary material 7 Distribution of pSc119.2 and pAs1 or pTa-535 (t) probes on chromosomes of Ae.comosasubsp.comosa and subsp.heldreichii Data type figure (TIF-file) Explanation note Distribution of pSc119.2 (green) and pAs1 (a-s) or pTa-535 (t) (red) probes on chromosomes of Ae.comosasubsp.comosa (a-j) and subsp. heldreichii (k-t): a – AE 115; b – AE 1254; c – AE 1256; d – AE 1257; e – AE 1258; f – AE 1259; g – AE 1377; h – K-3309; i – AE 1378; j – K-3857; k – K-3914; l – K-2432; m, n – K-1601; o – AE 117; p – K-4873; q – K-783; r – K-3897; s – K-3811; t – K-669. Translocated 1M^h^-6M^h^ chromosomes are arrowed. https://binary.pensoft.net/file/836919This dataset is made available under the Open Database License (http://opendatacommons.org/licenses/odbl/1.0/). The Open Database License (ODbL) is a license agreement intended to allow users to freely share, modify, and use this Dataset while maintaining this same freedom for others, provided that the original source and author(s) are credited.Ekaterina D. Badaeva, Violetta V. Kotseruba, Andnrey V. Fisenko, Nadezhda N. Chikida, Maria Kh. Belousova, Peter M. Zhurbenko, Sergei A. Surzhikov, Alexandra Yu. Dragovich

Supplementary material 8 Distribution of pTa794 and oligo-pTa71 or o-18S probes on chromosomes of Ae.comosasubsp.heldreichii and *Ae.crassa* Data type figure (TIF-file) Explanation note Distribution of pTa794 (red, a-c) and oligo-pTa71 (b) or o-18S (green, a, c) probes on chromosomes of Ae.comosasubsp.heldreichii (a, b) and *Ae.crassa* 6x, IG 131680 (c). a – c: Position of major NORs visualized with o-18S probe are shown with yellow arrows; position of major and minor NORs visualized with o-pTa71 probe are shown with red arrows. d – alignment of different variants of 18S rDNA fragments identified in *Ae.tauschii* genome using blast of NCBI SRA database (https://www.ncbi.nlm.nih.gov/sra). https://binary.pensoft.net/file/836920This dataset is made available under the Open Database License (http://opendatacommons.org/licenses/odbl/1.0/). The Open Database License (ODbL) is a license agreement intended to allow users to freely share, modify, and use this Dataset while maintaining this same freedom for others, provided that the original source and author(s) are credited.Ekaterina D. Badaeva, Violetta V. Kotseruba, Andnrey V. Fisenko, Nadezhda N. Chikida, Maria Kh. Belousova, Peter M. Zhurbenko, Sergei A. Surzhikov, Alexandra Yu. Dragovich

Supplementary material 9 List of *Ae.comosa* accessions and their origin Data type table (.docx file) https://binary.pensoft.net/file/836922This dataset is made available under the Open Database License (http://opendatacommons.org/licenses/odbl/1.0/). The Open Database License (ODbL) is a license agreement intended to allow users to freely share, modify, and use this Dataset while maintaining this same freedom for others, provided that the original source and author(s) are credited.Ekaterina D. Badaeva, Violetta V. Kotseruba, Andnrey V. Fisenko, Nadezhda N. Chikida, Maria Kh. Belousova, Peter M. Zhurbenko, Sergei A. Surzhikov, Alexandra Yu. Dragovich
