# Supplementary figures and images for: ﻿Intraspecific divergence of diploid grass Aegilopscomosa is associated with structural chromosome changes
Source: Comp Cytogenet. 2023 Apr 12;17:75–112. doi: 10.3897/CompCytogen.17.101008 (PMC10252141; doi:10.3897/CompCytogen.17.101008)

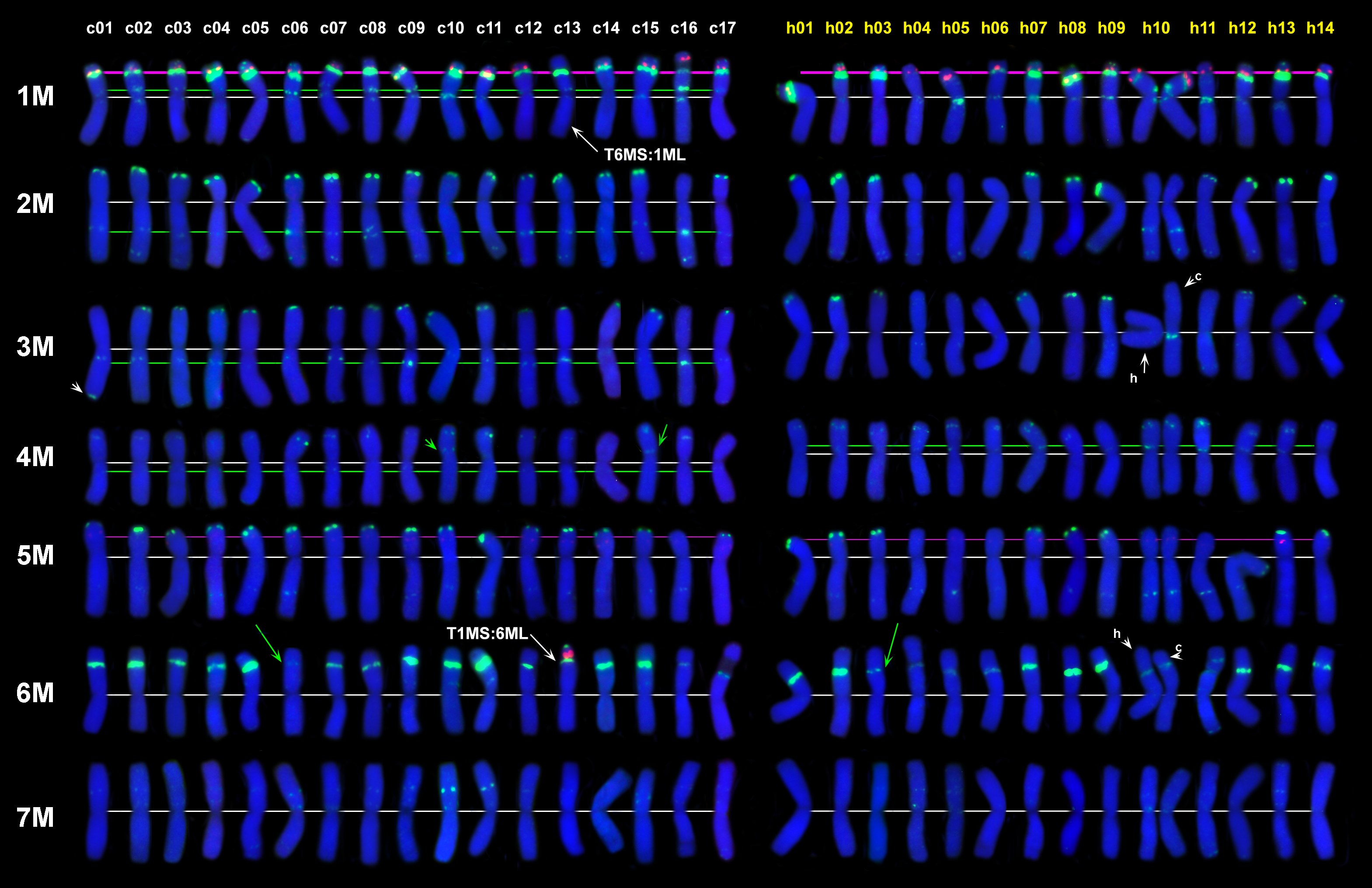

Supplement: Supplementary material 1 — Variation of hybridization patterns of pTa794 and o-18S or pTa71 rDNA probes [file comparative_cytogenetics-17--075_article-101008__-s001.tif]

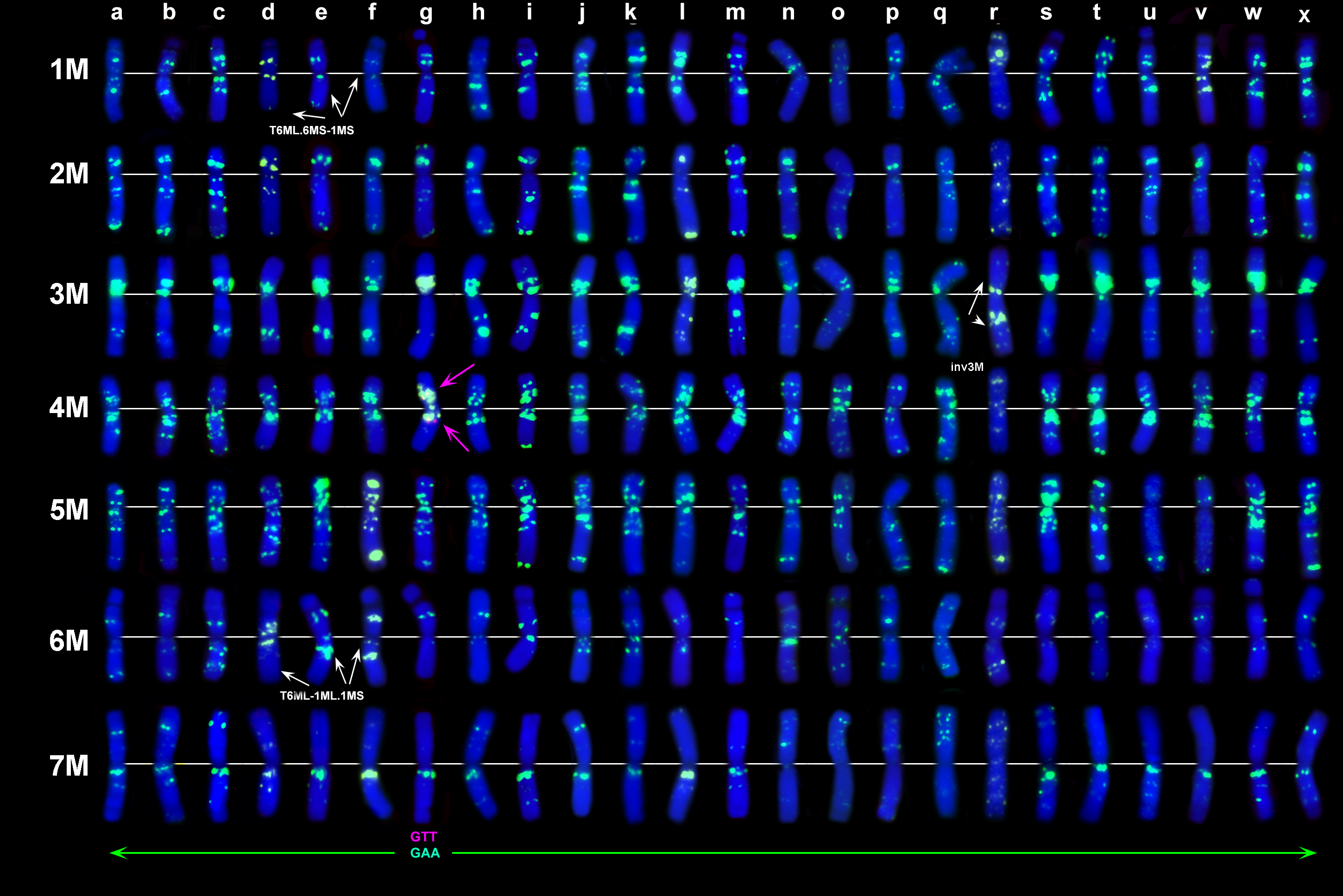

Supplement: Supplementary material 2 — Distribution of (GAA)10 microsatellite probe on chromosomes of different accessions of Ae.comosasubsp.comosa [file comparative_cytogenetics-17--075_article-101008__-s002.tif]

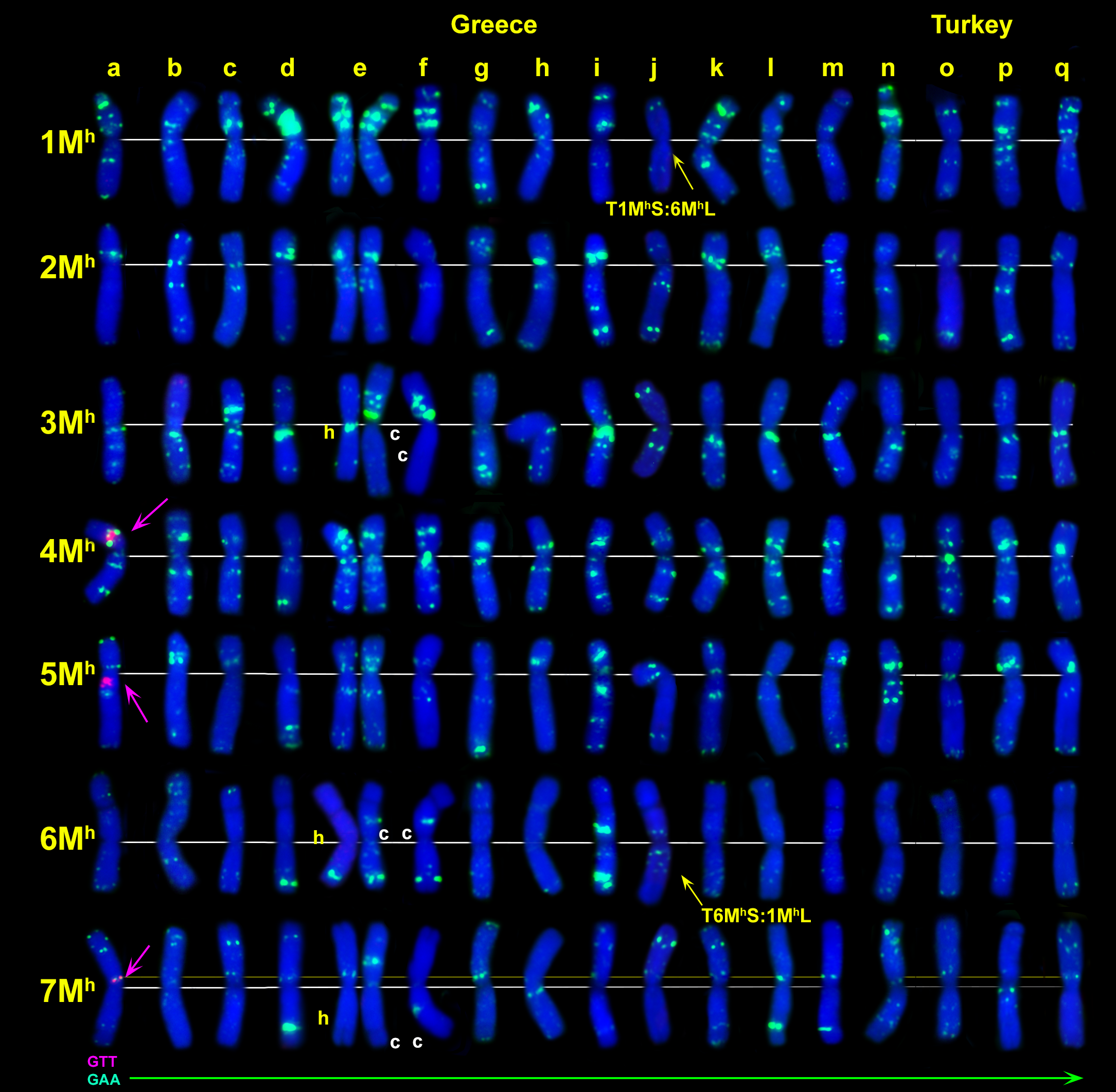

Supplement: Supplementary material 3 — Distribution of (GAA)10 microsatellite probe on chromosomes of different accessions of Ae.comosasubsp.heldreichii [file comparative_cytogenetics-17--075_article-101008__-s003.tif]

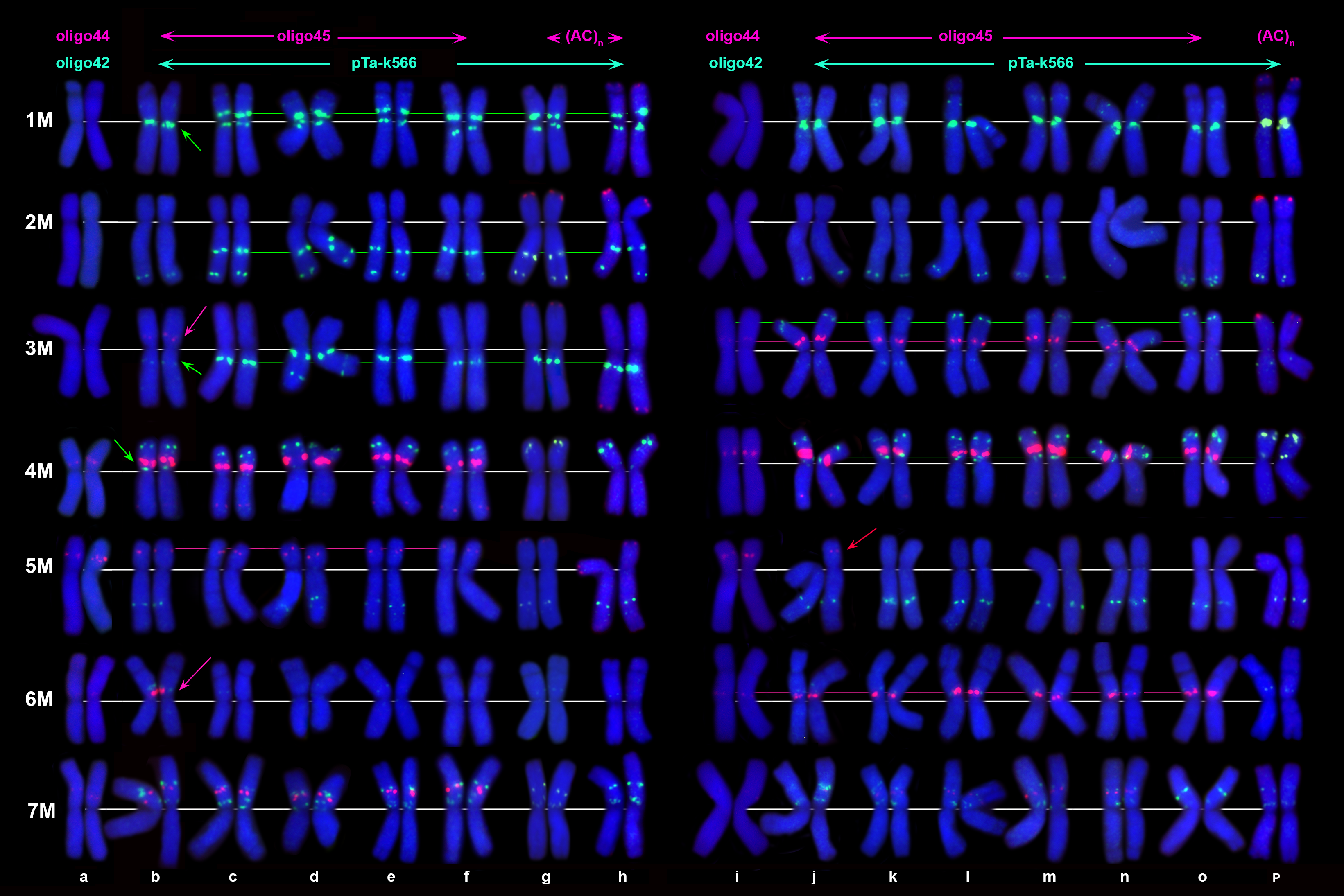

Supplement: Supplementary material 4 — Distribution of oligo-42, oligo-44, oligo-45, (AC)20, and pTa-k566 probes on chromosomes of Ae.comosasubsp.comosa and subsp.heldreichii [file comparative_cytogenetics-17--075_article-101008__-s004.tif]

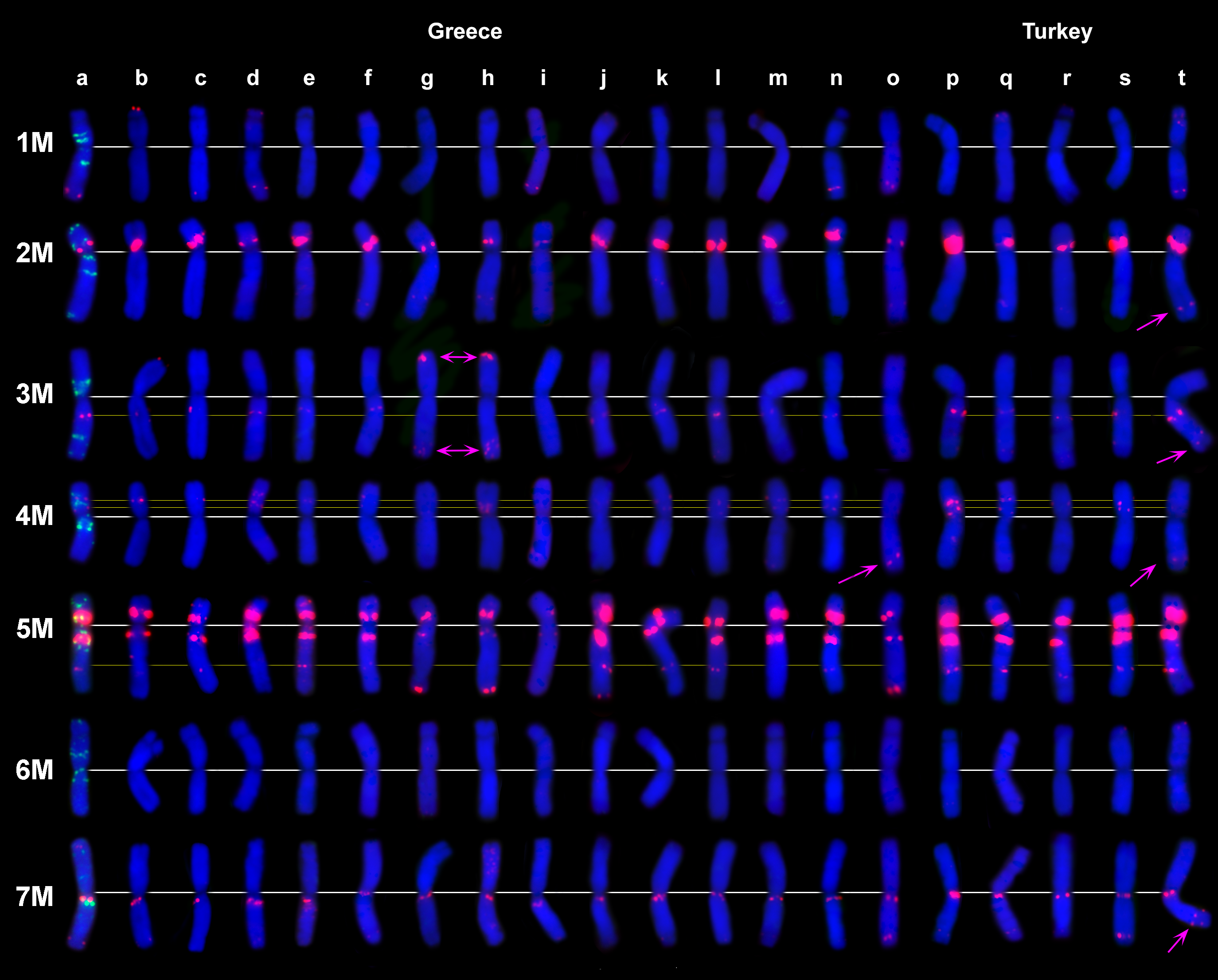

Supplement: Supplementary material 5 — Distribution of pTa-713 probe on chromosomes of different accessions of Ae.comosasubsp.comosa from Greece and Turkey [file comparative_cytogenetics-17--075_article-101008__-s005.tif]

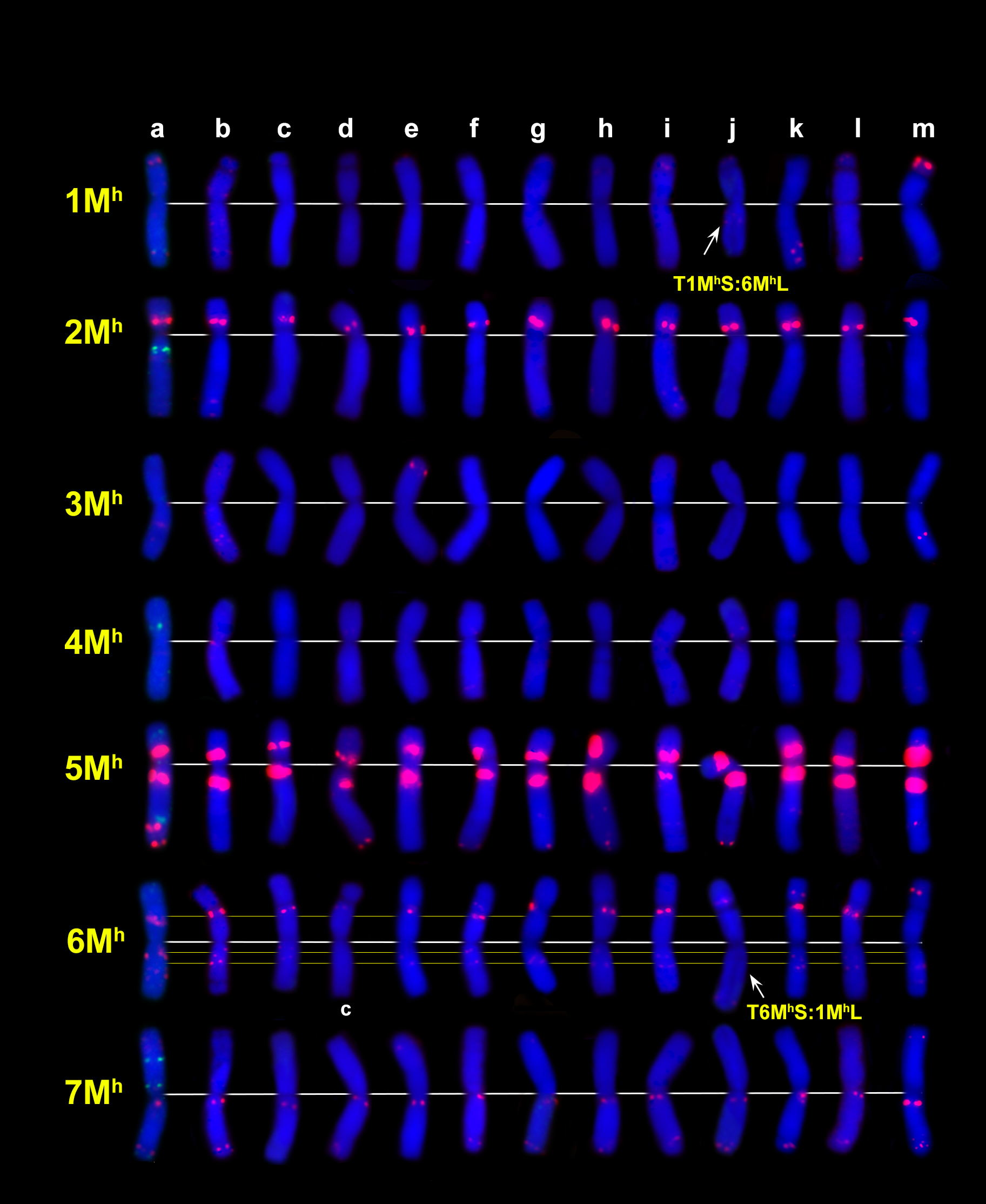

Supplement: Supplementary material 6 — Distribution of pTa-713 probe on chromosomes of different accessions of Ae.comosasubsp.heldreichii [file comparative_cytogenetics-17--075_article-101008__-s006.tif]

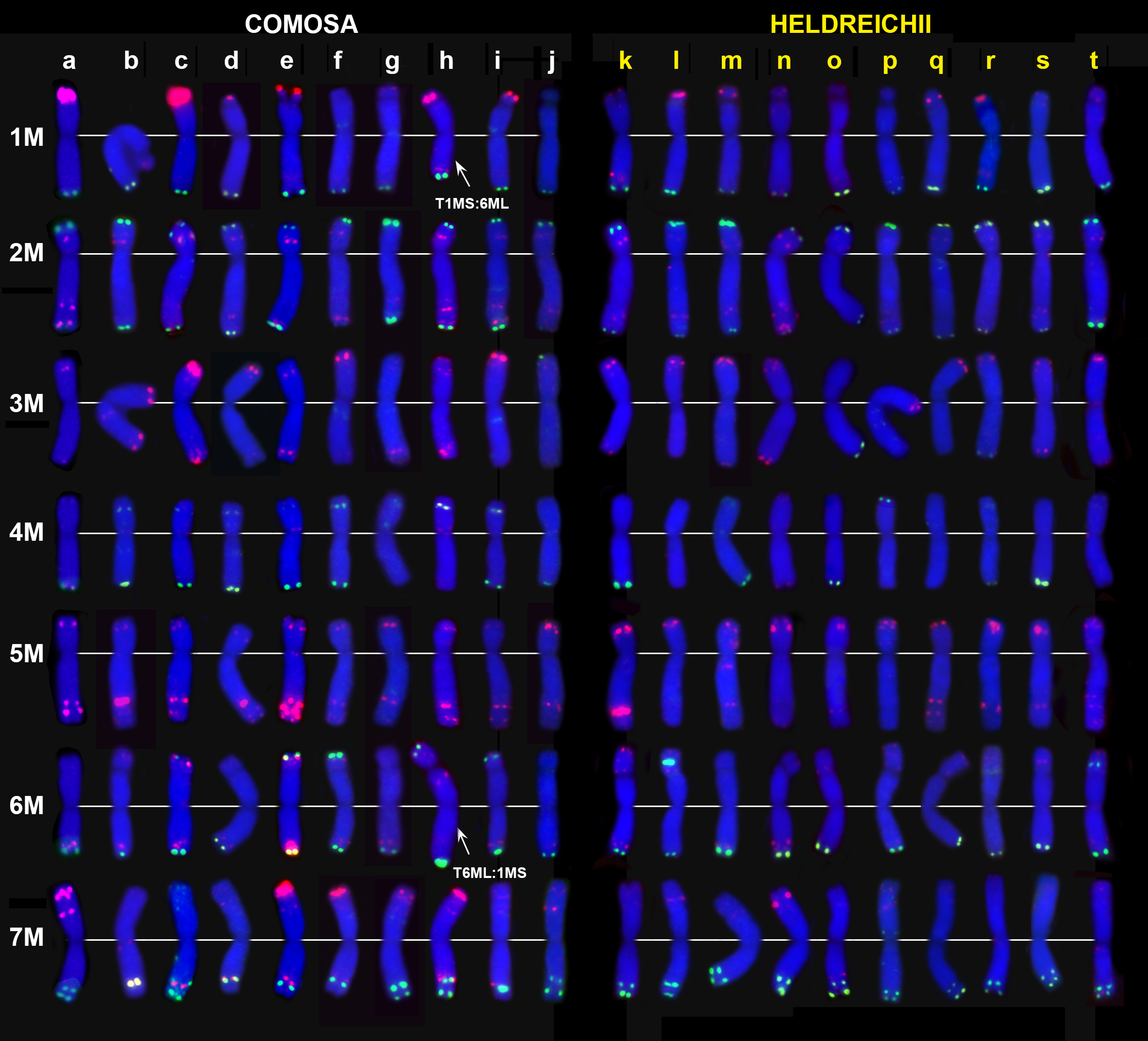

Supplement: Supplementary material 7 — Distribution of pSc119.2 and pAs1 or pTa-535 (t) probes on chromosomes of Ae.comosasubsp.comosa and subsp.heldreichii [file comparative_cytogenetics-17--075_article-101008__-s007.tif]

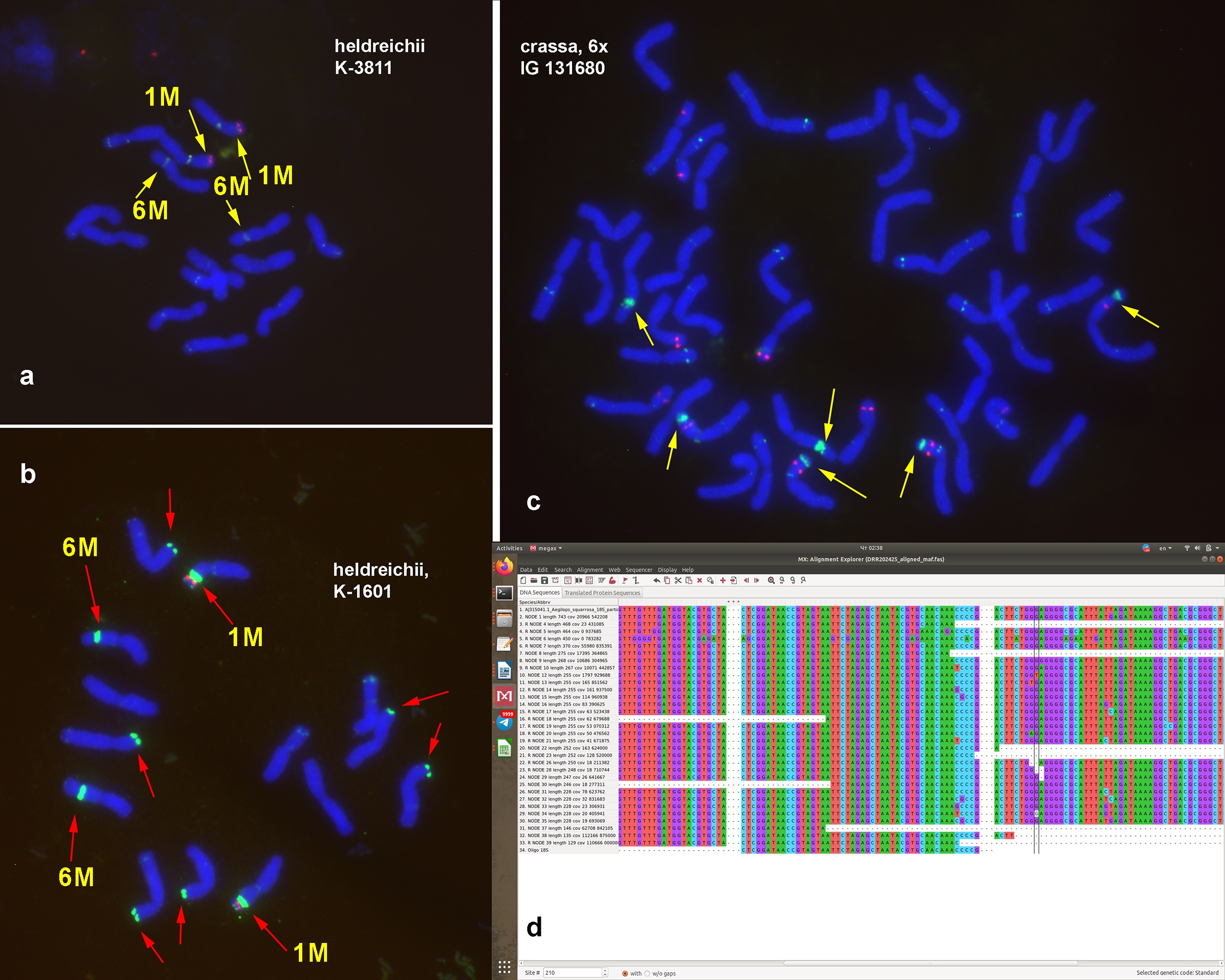

Supplement: Supplementary material 8 — Distribution of pTa794 and oligo-pTa71 or o-18S probes on chromosomes of Ae.comosasubsp.heldreichii and Ae.crassa [file comparative_cytogenetics-17--075_article-101008__-s008.tif]
