# Supplementary material for: ﻿Intraspecific divergence of diploid grass Aegilopscomosa is associated with structural chromosome changes
Source: Comp Cytogenet. 2023 Apr 12;17:75–112. doi: 10.3897/CompCytogen.17.101008 (PMC10252141; doi:10.3897/CompCytogen.17.101008)
Supplement: Supplementary material 9 — List of Ae.comosa accessions and their origin [file comparative_cytogenetics-17--075_article-101008__-s009.docx]

Supplementary Table 1. The list of *Ae. comosa* accessions and their origin

| No | accession # | Duplicates | Sub-species | Country of origin | Collection site | latitude (N) | longitude € | altitude (h), m |
| --- | --- | --- | --- | --- | --- | --- | --- | --- |
| 1 | AE 117 | K-641 | *heldreichii* | Greece | Peloponnes, Epidaurus | N 37 35 | E 23 04 | - |
| 2 | AE 783 | - | *heldreichii* | unknown | unknown | - | - | - |
| 3 | K-1601 | - | *heldreichii* | Greece | Central Greece and Evvoia, Near Panorama on northeast slopes of Pendeli | N 38 06 | E 23 54 | 413 |
| 4 | K-2272 | - | *heldreichii* | Greece | Central Greece and Evvoia, Near Panorama on northeast slopes of Pendeli | N 38 06 | E 23 54 | 413 |
| 5 | K-2432 | - | *heldreichii* | Greece | unknown | - | - | - |
| 6 | K-4873 | - | *heldreichii* | Greece | unknown | - | - | - |
| 7 | K-3804 | IG 107254; PI 551017 | *heldreichii* | Greece | Thessalia, 1.5km before junction to Achladea on Kalambaka-Grevena road, Trikala | N 39 83 | E 21 55 | - |
| 8 | K-3806* | IG 107256; PI 551022 | *heldreichii* | Greece | Macedonia, 1km before Kendia on Thessaloniki-Chalkidiki road, Thessaloniki | 40.7 | 22.7 | - |
| 9 | K-3811 | IG 107261; PI 551027 | *heldreichii* | Greece | Central Greece and Evvoia, Near Panorama on northeast slopes of Mt. Penteli, Attica. | N 38 07 | E 23 55 | 308 |
| 10 | K-3824 | IG 48597 | *heldreichii* | Turkey | Istanbul. Gebze-Istanbul road | N 38 23 | E 23 36 | - |
| 11 | K-3897* | IG 48591 | *heldreichii* | Turkey | Denizli. 6 km W Denizli | 37.8 | E 29 01 | - |
| 12 | K-3919 | IG 48602 | *heldreichii* | Greece | Epirus (Paramythia) | 39,4667 | E 20,5 | - |
| 13 | K-4498 | IG 107253; PI 551020 | *heldreichii* | Greece | 1km SE of Zarkos, Trikala | N 39 07 | E 22 07 60 | 100 |
| 14 | K-669 | - | *heldreichii* | Greece | Peloponnes, around Corinth | N 37 56 | E 22 57 | - |
| 15 | К-3914 | IG 48597; PI 551017 | *heldreichii* | Turkey | Istanbul. Gebze-Istanbul road | N 40 57 04 | E 29 18 47 | - |
| 16 | K-3809 | IG 107259; PI 551025 | *F1 comosa x heldreichii* | Greece | Central Greece and Evvoia, Near Dionissos northern foothills of Mt. Penteli Attica | N 38 04 60 | E 23 54 | - |
| 17 | AE 115 | K-642; TA2733 | *comosa* | Greece | Peloponnes, 10 km S of Corinth | N 37 51 | E 22 59 | - |
| 18 | AE 1254 | - | *comosa* | Greece | Kykladen, Paros | N 37 04 59 | E 25 08 59 | - |
| 19 | AE 1256 | - | *comosa* | Greece | Kykladen, Andiparos | 37.033056 | 25.083056 | - |
| 20 | AE 1257 | - | *comosa* | Greece | Kykladen, Folegandros | 36.633056 | 24.899722 | - |
| 21 | AE 1258 | - | *comosa* | Greece | Kykladen, Folegandros | 36.633056 | 24.899722 | - |
| 22 | AE 1259 | - | *comosa* | Greece | Kykladen, Sifnos | N 37 00 | 24.666389 | - |
| 23 | AE 1260 | - | *comosa* | Greece | Kykladen, Sifnos | N 37 00 | 24.666389 | - |
| 24 | AE 1376 | - | *comosa* | Greece | unknown | - | - | - |
| 25 | AE 1377 | - | *comosa* | Greece | unknown | - | - | - |
| 26 | AE 1378 | - | *comosa* | Greece | unknown | - | - | - |
| 27 | K-3308 | IG 47132; PI 542175 | *comosa* | Turkey | Asagisamli-Denizli 500m from junc. on Pamukkale road | 37,85 | 29,15 | 180 |
| 28 | K-3309 | IG 48596 | *comosa* | Turkey | Izmir. 18 km N Menemen | 38,15 | 27,0333 | - |
| 29 | K-3780 | IG 47020; PI 486234 | *comosa* | Turkey | 10 km N Kusadasi | 37.9167 | 27.2833 | 130 |
| 30 | K-3781 | IG 47087 | *comosa* | Turkey | from AARI Izmir (K-181) | 38.6 | 27.0667 | - |
| 31 | K-3787 | IG 48591 | *comosa* | Turkey | Denizli. 6 km W Denizli | 37.8 | 29.0167 | - |
| 32 | K-3808 | IG 107258; PI 551024 | *comosa* | Greece | Central Greece and Evvoia, 2 km E of Politia, Attica | 38.083333 | 23.883333 | - |
| 33 | K-3810 | IG 107260; PI 551026 | *comosa* | Greece | Central Greece and Evvoia, Near Panorama on northeast slopes of Mt. Penteli, Attica. | 38.116667 | 23.916667 | 308 |
| 34 | K-3819 | IG 107262; PI 551028 | *comosa* | Greece | Central Greece and Evvoia, 3km W of Rafina on Marathon-Athens road, Attica | 38.033333 | 23.983333 | 100 |
| 35 | K-3820 | IG 107263; PI 551029 | *comosa* | Greece | Central Greece and Evvoia, 1 km from Pikermion on road to Athens | N 38 00 | E 23 56 | 150 |
| 36 | K-3857 | IG 107265 | *comosa* | Greece | Central Greece and Evvoia, 2 km N of Skimatarion on road to Chalkidiki | 38.366667 | 23.583333 | 200 |
| 37 | K-3920* | IG 48603 | *comosa* | Turkey | Antalya, 13 km E Yarpuz | N 37 07 | E 31 52 | 40 |
